# Measuring kinetics and metastatic propensity of CTCs by blood exchange between mice

**DOI:** 10.1038/s41467-021-25917-5

**Published:** 2021-09-28

**Authors:** Bashar Hamza, Alex B. Miller, Lara Meier, Max Stockslager, Sheng Rong Ng, Emily M. King, Lin Lin, Kelsey L. DeGouveia, Nolawit Mulugeta, Nicholas L. Calistri, Haley Strouf, Christina Bray, Felicia Rodriguez, William A. Freed-Pastor, Christopher R. Chin, Grissel C. Jaramillo, Megan L. Burger, Robert A. Weinberg, Alex K. Shalek, Tyler Jacks, Scott R. Manalis

**Affiliations:** 1 Department of Electrical Engineering and Computer Science, Massachusetts Institute of Technology, Cambridge, MA USA; 2 David H. Koch Institute for Integrative Cancer Research, Massachusetts Institute of Technology, Cambridge, MA USA; 3 Harvard-MIT Department of Health Sciences and Technology, Institute for Medical Engineering and Science, Massachusetts Institute of Technology, Cambridge, MA USA; 4 Department of Oncology, Hematology and Bone Marrow Transplantation with Section Pneumology, Hubertus Wald Comprehensive Cancer Center Hamburg, University Medical Center Hamburg-Eppendorf, Martinistrasse 52, Hamburg, Germany; 5 Department of Tumor Biology, Center of Experimental Medicine, University Medical Center Hamburg-Eppendorf, Hamburg, Germany; 6 Department of Mechanical Engineering, Massachusetts Institute of Technology, Cambridge, MA USA; 7 Department of Biology, Massachusetts Institute of Technology, Cambridge, MA USA; 8 Department of Biomedical Engineering, Wentworth Institute of Technology, Boston, MA USA; 9 Department of Biological Engineering, Massachusetts Institute of Technology, Cambridge, MA USA; 10 Department of Medical Oncology, Dana-Farber Cancer Institute, Boston, MA USA; 11 Whitehead Institute for Biomedical Research, Cambridge, MA USA; 12 Department of Chemistry, Massachusetts Institute of Technology, Cambridge, MA USA; 13 Institute for Medical Engineering and Science, Massachusetts Institute of Technology, Cambridge, MA USA; 14 Broad Institute of MIT and Harvard, Cambridge, MA USA; 15 Ragon Institute of MGH, MIT and Harvard University, Cambridge, MA USA; 16 Department of Immunology, Massachusetts General Hospital, Boston, MA USA; 17 Ludwig Center at MIT’s Koch Institute for Integrative Cancer Research, Cambridge, MA USA

**Keywords:** Cancer models, Biophysical methods, Lab-on-a-chip, Cancer models

## Abstract

Existing preclinical methods for acquiring dissemination kinetics of rare circulating tumor cells (CTCs) en route to forming metastases have not been capable of providing a direct measure of CTC intravasation rate and subsequent half-life in the circulation. Here, we demonstrate an approach for measuring endogenous CTC kinetics by continuously exchanging CTC-containing blood over several hours between un-anesthetized, tumor-bearing mice and healthy, tumor-free counterparts. By tracking CTC transfer rates, we extrapolated half-life times in the circulation of between 40 and 260 s and intravasation rates between 60 and 107,000 CTCs/hour in mouse models of small-cell lung cancer (SCLC), pancreatic ductal adenocarcinoma (PDAC), and non-small cell lung cancer (NSCLC). Additionally, direct transfer of only 1−2% of daily-shed CTCs using our blood-exchange technique from late-stage, SCLC-bearing mice generated macrometastases in healthy recipient mice. We envision that our technique will help further elucidate the role of CTCs and the rate-limiting steps in metastasis.

## Abstract (web summary)

Current methods for acquiring dissemination kinetics of rare circulating tumor cells (CTCs) that form metastases have several limitations. Here, the authors show an approach for measuring endogenous CTC kinetics by continuously exchanging CTC-containing blood between un-anesthetized, tumor-bearing mice and healthy, tumor-free counterparts.

## Introduction

Circulating tumor cells (CTCs)—cells shed into the bloodstream from primary and metastatic tumor deposits—represent the intermediary component of the metastatic cascade. Measuring their intravasation rate and half-life time in the circulatory system, which together govern their blood levels, has been an important step towards elucidating the kinetics of their seeding of distant tissues and the subsequent outgrowth of metastatic colonies. Traditionally, the fate of tumor cells has been examined by injecting tumor cell lines intravenously into animal models (primarily mice, rats, and rabbits)^1–4^. By analyzing terminal blood and other major organs from multiple animals at different time points post inoculation, these initial studies suggested extremely short half-life times in circulation (less than 1 s)^3,5^. The intravenously injected cells seemed to arrest in the capillaries of the first organs they encountered almost immediately after injection, but their subsequent proliferation into secondary lesions was influenced by host-tumor cell interactions operating within specific organs. Although these studies established the basis of the “seed and soil” hypothesis^6–8^, these methods have not been amenable to the study of endogenously-generated CTCs that originate from solid primary tumors, which would be expected to exhibit different physiology than CTCs derived from established cell lines. Additionally, previous methods^9–19^ have not yet provided direct measures of the intravasation rate for CTCs.

Beyond the circulatory dynamics, a direct, functional assessment of the intrinsic propensity of CTCs to proliferate in the parenchyma of distant organs is important for identifying the biological properties of these metastasis-initiating cells. Current preclinical approaches to address this aspect rely on either murine cell lines or CTCs isolated from patient blood samples ex vivo prior to their injection into immunocompromised animals^20–25^. While these various studies presented different approaches for growing tumors in laboratory animals to study metastasis or explore different potential therapeutic options, newer preclinical methods utilizing immunocompetent mice and requiring less ex vivo manipulation of CTCs are likely to facilitate a deeper understanding of the role CTCs play in metastasis.

Here, we present a blood-exchange method, between pairs of un-anesthetized mice, for studying the kinetics of endogenous CTCs in the context of surrounding stroma and a fully functional host immune system. We apply our method to an autochthonous, genetically engineered mouse model (GEMM) of small-cell lung cancer (SCLC), which is characterized by high CTC levels in the blood, in addition to a GEMM of non-small-cell lung cancer (NSCLC) and various models of pancreatic ductal adenocarcinoma (PDAC), which are characterized by significantly lower CTC levels. Unlike parabiosis, our method does not require a permanent surgical connection between the vasculatures of the two mice. Instead, blood is temporarily exchanged for several hours through the implanted catheters. The blood-exchange method we describe here enables a series of experiments that can answer fundamental questions about the relationship between CTC characteristics and metastasis.

## Results

### Blood exchange for direct CTC kinetics studies

To measure the CTC half-life time and generation (intravasation) rate, we utilized a syngeneic, non-tumor-bearing, “healthy” mouse (HM) as a recipient for CTCs generated by a tumor-bearing mouse (TBM). Three murine tumor models with varying CTC levels were characterized. Primary tumors in the autochthonous SCLC model are initiated by delivering Cre-expressing virus to the murine lung epithelium, leading to the deletion of the *Trp53*, *Rb1*, and *Pten* (collectively known as PRPten) tumor suppressor genes^26^ and activation of a tdTomato allele that engenders red fluorescence in all tumor cells, including derived CTCs. SCLC TBMs survive for approximately six months post tumor initiation, with detectable primary (lung) and metastatic (liver) tumors starting at approximately five months post tumor initiation. PDAC models used in this study included both an autochthonous mouse model initiated via retrograde pancreatic duct delivery of Cre-expressing virus, which activates a *Kras* G12D mutation, deletes *Trp53*, and activates a tdTomato allele^27^, and two organoid-based orthotopic models (one with and one without the expression of a defined neoantigen). The NSCLC model was initiated through viral Cre-mediated deletion of *Trp53*, activation of a *Kras* G12D mutation, and activation of a tdTomato allele^28–30^.

**Fig. 1:**
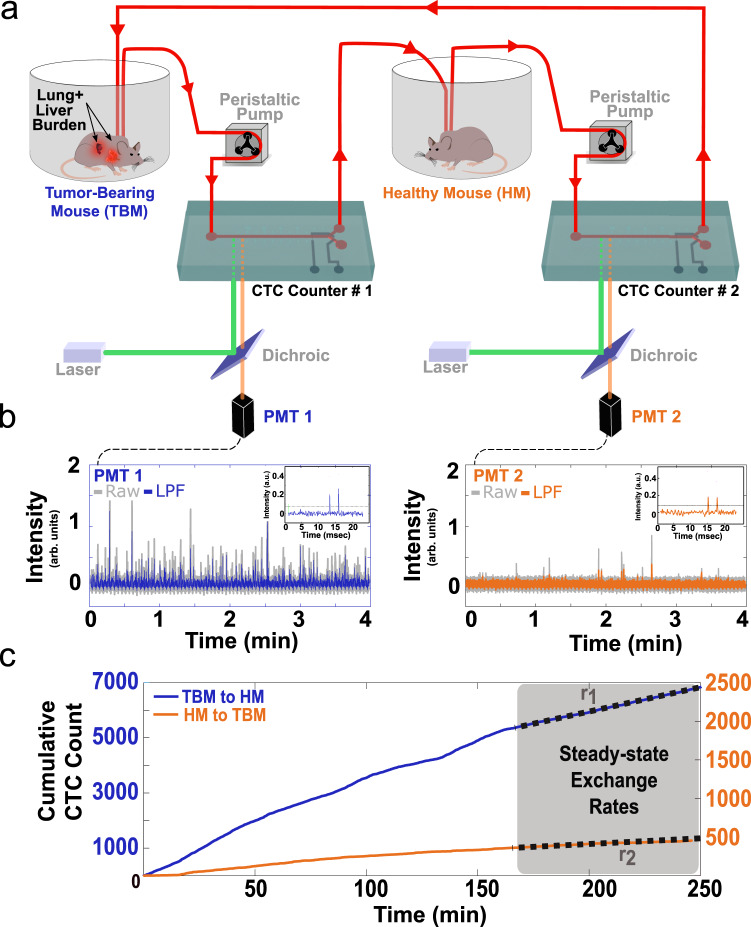
A blood-exchange method for direct CTC kinetics studies. **a** Schematic demonstrating the blood exchange method in which the circulatory systems of two mice (one tumor-bearing mouse (TBM) and one healthy mouse (HM)) are connected in a closed-loop through two CTC counters. Two peristaltic pumps set to identical flow rates push the blood around the system and through the CTC counters. For each CTC counter, a series of two laser lines is used to excite the flowing genetically-fluorescent CTCs (see Supplementary Figs. 1−3 for more details). The emitted signal is directed through a dichroic filter toward a photomultiplier tube (PMT) for detection. **b** PMT raw and lowpass-filtered (LPF) spectra demonstrate a series of detected CTCs by each of the two CTC counters’ PMTs (PMT 1 = CTC counter 1 (blue), PMT 2 = CTC counter 2 (orange)). The inset demonstrates the LPF dual-peak configuration created when each CTC passes under the two laser lines projected across the flow channel. **c** Cumulative CTC counts over time from the two CTC counters demonstrate a higher injection rate (blue trajectory with left *Y*-axis) compared to return rate (orange trajectory with the right *Y*-axis). Gray-shaded regions represent the assumed steady-state time interval over which the exchange rates were estimated.

In our experimental setup (Fig. 1a), CTC-containing blood is exchanged between a TBM (the example shown in the figure depicts a five to six-month post-initiation SCLC TBM) and a HM counterpart of the same sex and genotype, and similar age and weight. The exchanged blood is monitored in real-time through two CTC Counters, as it is pumped at a flow rate of 60 µL/min in a closed-loop manner (Supplementary Figs. 1 and 2a). Within each CTC Counter, fluorescent CTCs are excited using a 532 nm dual*-*excitation laser beam configuration focused across the microfluidic blood-flow channel near the inlet of the device (Supplementary Figs. 1 and 3). The resulting dual emission peaks from the photomultiplier tube (PMT) are recorded for offline peak analysis and validation purposes^31^ (Fig. 1b). Although our CTC Counters do not currently have the capability of distinguishing between single cells and clusters, fluorescent microscopy analyses of terminal blood samples collected from late-stage SCLC-bearing mice indicate that the majority of fluorescent (tdTomato+) cellular events in the bloodstream result from single cells.

To provide continued access to the circulatory system, mice undergo a cannulation surgery to externalize two catheters from the left carotid artery and the right jugular vein. Continuous blood flow between the two un-anesthetized mice is controlled by two peristaltic pumps and can be executed over several hours until steady-state CTC exchange rates are measured. The relatively high heart rate in mice (400−600 beats per minute resulting in high cardiac outputs of approximately 20 mL/min^32^) and low total circulating blood volume (approximately 1.5−2 mL) allow us to assume that CTCs are uniformly distributed in the bloodstream of each mouse, when sampled continuously at the much lower volumetric flow rate of 60 µL/min between the two mice. This assumption was validated empirically by observing similar CTC concentrations in flowing blood during real-time scans and terminal blood samples collected by cardiac puncture from four SCLC-bearing animals (Supplementary Table 1).

In more detail, CTCs drawn from the TBM’s carotid artery catheter pass through the first, in-line CTC counter (labeled as “CTC Counter #1” in Fig. 1a and Supplementary Fig. 2a) for real-time enumeration prior to their introduction into the jugular vein catheter of the HM. The total transport time of individual CTCs within the tubing from one circulatory system to the other is approximately 2 min at the 60 µL/min flow rate, during which minimal losses were observed (see “Methods” and Supplementary Fig. 2b). CTCs that remain in the circulation of the HM are detected and counted by the second CTC counter (“CTC Counter #2” in Fig. 1a and Supplementary Fig. 2a), prior to their return to the right jugular vein of the TBM. Once the raw PMT data are processed for validation of CTC counts (Fig. 1b), the cumulative counts over time from each CTC counter are then plotted to extract the CTC exchange rates necessary for calculating the half-life time and the generation rate of CTCs by the primary tumor. Figure 1c demonstrates an example of the cumulative CTC count over time plot for one of these blood-exchange experiments, in which the HM received approximately 7,000 SCLC CTCs over the course of 4 h and returned approximately 500 CTCs to the TBM during this time period. The last 90 min of the experiment is used as a steady-state interval, during which exchange rates between the mice (*r*_1_ and *r*_2_) are extracted to calculate the real-time kinetics.

### Modeling the blood-exchange kinetics

**Fig. 2:**
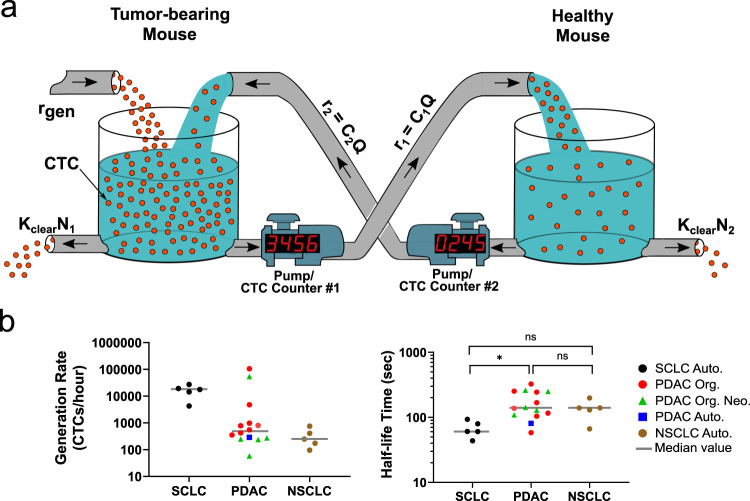
An analytical model for extracting CTC generation rate and half-life time in blood. **a** A visual representation of the relevant parameters of the blood exchange technique to solve for the generation rate and the half-life time of CTCs. The circulatory system of each mouse is represented as a well-mixed container of red spheres (CTCs). In the TBM (left tank) CTCs enter the circulation from the tumor microenvironment at a rate equal to ***r***_**gen**_. CTC clearance out of the circulation is represented by a hole at the bottom of each container with a clearance rate of ***K***_**clear**_ **×** ***N****.* Pumps with counters represent the CTC counter systems and their peristaltic pumps that transfer the CTC-containing blood at rates equal to ***C × Q***. *r*: rate of CTC transfer. *C*: concentration. *Q*: flow rate. *K*_clear_: first-order clearance coefficient. *N*: total number of CTCs in each mouse. **b** Scatter plots for the calculated CTC generation rates and the half-life times of each of the different small-cell lung cancer (SCLC), pancreatic ductal adenocarcinoma (PDAC), and non-small-cell lung cancer (NSCLC) blood-exchange experiments (*p < 0.05 (*p* = 0.0136), Kruskal−Wallis nonparametric test with Dunn’s sample pairs analysis). For SCLC, *n* = 5 biologically independent experiments. For PDAC, *n* = 8, 5, and 1 biologically independent experiments, respectively, for Org, Org. Neo., and Auto. For NSCLC, *n* = 5 biologically independent experiments. Auto autochthonous. Org. organoid. Neo neoantigen. ns not significant.

Analysis of the numbers of CTCs exchanged between the tumor-bearing and healthy mice (in both directions) allows us to monitor the instantaneous concentration of CTCs in each mouse, which in turn enables us to estimate the rate at which CTCs are being shed from the tumor, and the rate at which they are cleared from the circulation thereafter. To estimate the CTC generation rate and half-life time, we fit our data to a simple model in which CTCs (represented by the red spheres in Fig. 2a) are generated from the tumor microenvironment at a constant rate $({r}_{{{{{{{\mathrm{gen}}}}}}}})$ in the TBM and are cleared from the circulation of both mice by either lodging into capillaries across the different organs or by elimination through cell death (clearance represented by the holes at the bottom of the tanks in Fig. 2a).

We assume first-order kinetics where CTCs remain in circulation for a half-life time of ${t}_{1/2}$, or, equivalently, are cleared with rate constant ${K}_{{{{{{\rm{clear}}}}}}}={{{{{\rm{ln}}}}}}(2)/{t}_{1/2}$. The differential equations describing how the number of CTCs in each mouse change with time are:

1 $$\frac{{{{d}}N}}{{{{d}}t}}={{{{{\rm{rate}}}}}}\; {{{{{\rm{of}}}}}}\; {{{{{\rm{CTCs}}}}}}\; {{{{{\rm{in}}}}}}-{{{{{\rm{rate}}}}}}\; {{{{{\rm{of}}}}}}\; {{{{{\rm{CTCs}}}}}}\; {{{{{\rm{out}}}}}}$$

2 $${{{{{\rm{Tumor}}}}}}{\mbox{-}}{{{{{\rm{bearing}}}}}}\,\,{{{{{\rm{mouse}}}}{\!\!}:}}\,\frac{{{d}}{n}_{1}}{{{{d}}t}}=-{C}_{1}Q+{C}_{2}Q-{K}_{{{{{{\rm{clear}}}}}}}{n}_{1}\left(t\right)+{r}_{{{{{{\rm{gen}}}}}}}$$

3 $${{{{{\rm{Healthy}}}}}}\,\,{{{{{\rm{mouse}}}}}}{\!\!}:\frac{{{d}}{n}_{2}}{{{{d}}t}}={C}_{1}Q-{C}_{2}Q-{K}_{{{{{{\rm{clear}}}}}}}{n}_{2}(t)$$

where $Q$ is the constant volumetric flow rate of the pumps exchanging the blood of the mice, $V$ is the total blood volume of each mouse, and $C\left(t\right)={n}_{1}(t)/V$ and ${C}_{2}\left(t\right)={n}_{2}(t)/V$ are the concentrations of CTCs in the tumor-bearing and healthy mice, respectively.

Each CTC counter (each represented by a pump with a digital counter in Fig. 2a) measures the *rate* at which CTCs pass from one mouse to another, which we assume to be proportional to the concentration of CTCs in the blood: ${r}_{1}(t)={C}_{1}(t)Q$ and ${r}_{2}(t)={C}_{2}(t)Q$ for the tumor-bearing-to-healthy CTC counter #1 and the healthy-to-tumor-bearing CTC counter #2, respectively. As discussed above, this “well-mixed” circulatory system assumption is justified, since the cardiac output and small total blood volume of a mouse results in approximately 50-fold greater flow rate of blood within a mouse due to its baseline circulation than the blood exchange flow rate between the mice of 60 μL/min.

Given measurements of ${r}_{1}$and ${r}_{2}$, the steady-state rates at which CTCs pass through both CTC counters, we can estimate the CTC generation rate and half-life in the circulation as:

4 $${r}_{{{{{{\bf{g}}}}}}{{{{{\bf{e}}}}}}{{{{{\bf{n}}}}}}}=({r}_{1}-{r}_{2})\left(1+\,\frac{{r}_{1}}{{r}_{2}}\right)[{{{{{\rm{CTCs}}}}}}/\,{{\min }}\,]$$

5 $${t}_{1/2}=\frac{(V/Q){{{{\mathrm{ln}}}}}(2)}{{r}_{1}/{r}_{2}-1}[\,{{\min }}\,]$$

To validate this model, we performed five SCLC, 14 PDAC, and five NSCLC blood-exchange experiments between pairs of compatible tumor-bearing mice and healthy, immunocompetent counterparts. At the beginning of each experiment, we performed an initial scan (for at least 10 min) in which each mouse’s blood was scanned separately to ensure proper CTC counter functionality, reliable CTC detection, and stable blood flow from the carotid artery through the CTC counter and back into the jugular vein. Afterwards, the blood-exchange process was executed for at least two hours to allow for a sufficient time period for the CTC exchange rates to stabilize. During the five SCLC blood exchange experiments, the total number of CTCs transferred from the TBMs to the HMs varied from 2,000 to 8,000 CTCs (blue trajectories in Supplementary Fig. 4a); 3−7% of these CTCs were returned from the HM to the TBM (orange trajectories in Supplementary Fig. 4a). In the PDAC blood exchange experiments, between 150 and 18,000 CTCs were transferred from the TBMs to the HMs and between 15 and 6,000 were returned. In the NSCLC experiments, 60−120 CTCs were transferred from the TBM to the HM, and 10−20 were returned.

In order to extract the steady-state exchange rates from the empirical data, a best fit line was applied to the cumulative count trajectories during the final interval of the blood-exchange experiments. During this interval, changes in CTC counts over time in each mouse (${{{{{{\mathrm{d}}}}}}n}/{{{{{{\mathrm{d}}}}}}t}$) were approximately zero (i.e., *r*_1_ and *r*_2_ are roughly constant). CTC exchange rates were assessed from different steady-state intervals (30, 45, 60, 75, and 90 min) to extrapolate the average and uncertainty (error) values for *r*_gen_ and *t*_1/2_ parameters for SCLC, PDAC, and NSCLC blood-exchange experiments (Fig. 2b; “Methods”). These *r*_1_ and *r*_2_ estimates are shown in Supplementary Table 2.

The CTC generation rate varied by several orders of magnitude both within and across the cancer models (Fig. 2b, and Supplementary Table 3), in part because the tumors were at a wide range of developmental stages at the times the blood exchanges occurred. For example, the late-stage SCLC TBMs in our study (five to six months post tumor initiation), with well-established primary lung and metastatic liver burden (Supplementary Fig. 4b), had CTC generation rates between 4,000 and 27,000 CTCs/hour. CTC generation rates in 12 of the 14 PDAC-bearing animals ranged from 65 to 6,000 CTCs/hour, with two PDAC tumor-bearing animals generating over 50,000 CTCs/hour. The NSCLC CTC generation rates ranged from 100 to 800 CTCs/hour. By assessing the total number of tumor cells at the primary site for each model (see “Methods”), it was possible to estimate the fraction of total number of tumor cells shed per day as well as the number of cells shed per gram of tumor tissue per day. The estimates of percentage of total tumor cells shed per day and number of tumor cells shed per gram of tumor are 0.07% or 700,000 cells per gram per day for SCLC-bearing animals; 0.004% or 40,000 cells per gram per day for PDAC-bearing animals; and 0.002% or 20,000 cells per gram per day, respectively, for NSCLC-bearing animals. In contrast to the CTC generation rate, the half-life time, ranging from 40 to 260 s, was more consistent both within and across the cancer models (Fig. 2b and Supplementary Table 3). Collectively, these results demonstrate the utility of the blood-exchange technique for measuring CTC kinetics in a variety of preclinical models with a range of CTC concentrations.

### Model cell line circulatory kinetics differ from CTCs

**Fig. 3:**
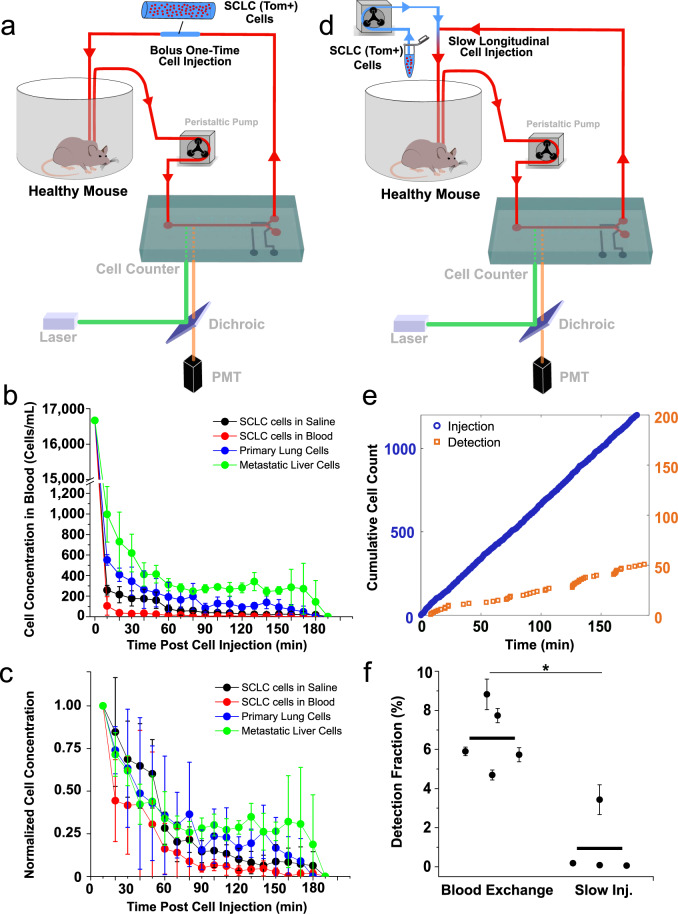
Comparison of CTC circulatory kinetics to SCLC cell line. **a** Schematic demonstrating the bolus cell-injection experiment configuration. A short segment of tubing filled with approximately 40 µL of blood or saline containing 25,000 cells of either SCLC cell line, dissociated primary tumor cells, or dissociated liver metastatic tumor cells is added to the blood return line at the beginning of the experiment for a direct injection of its contents into the circulation within approximately 1 min. Clearance kinetics plots representing the real-time concentration (**b**) and the normalized concentration to the initial (first 10 min) detected concentration (**c**). For each group, *n* = 3 biologically independent experiments. Data are presented as mean ± standard deviation. **d** Schematic demonstrating the slow cell-line injection experiment configuration. A second peristaltic pump is used to slowly infuse cell-containing saline into the circulation at a flow rate of 2−3 µL/min, with total injected cells mimicking that of the CTC blood exchange experiments. **e** Cumulative injection (blue) and detection (orange) cell counts over time. **f** Detection fraction plot representing the average fraction of total detected cells to the total injected cells in the last 30, 45, and 60 min of five blood-exchange experiments and four slow-injection experiments. *N* = 5 biologically independent blood exchange experiments and *n* = 4 biologically independent slow injection experiments. All values are represented as mean ± standard deviation. (*p < 0.05 (*p* = 0.016), two-sided Mann−Whitney−Wilcoxon non-parametric test).

In order to compare our observed CTC kinetics to traditional studies^2,4^, bolus injections of SCLC cell lines were carried out. We collected 25,000 tdTomato-expressing viable cells prepared from an established murine SCLC cell line that had been generated from murine SCLC tumors isolated from Trp53^fl/fl^; Rb1^fl/fl^; Pten^fl/fl^; Rosa26^LSL-tdTomato/LSL-Luciferase^ mice and propagated in vitro^33^. These cells were suspended in 40 µL of either saline or blood and were injected intravenously via the inserted cannula, followed by three-hour scans of the live mouse’s blood with the CTC counter (Fig. 3a). In contrast to the behavior of endogenous CTCs, described above, we observed that nearly 98% of the injected cells cleared from the circulation within seconds (Fig. 3b, c). Furthermore, when we repeated the bolus injection experiments using tumor cells that were freshly-harvested and dissociated from primary and metastatic tumors from late-stage, autochthonous SCLC-bearing mice, we observed a similar rapid clearance of these cells from the circulation, suggesting that the observed phenomenon was not an artifact of using in vitro cultured tumor cell lines. Nonetheless, across all four cell injection experiments, we observed that a small percentage (1−2%) of the injected cells remained detectable in the blood for several hours (Fig. 3b, c). These observations provided an initial indication that direct bolus injection of tumor cells into the venous circulation is likely to misrepresent the true dynamics of CTC persistence in circulation.

To examine whether the observed kinetics of clearance following intravenous injection were influenced by the rate at which tumor cells were introduced into circulation, we mimicked the continuous, blood-exchange-based injection rate by using a slow, cell-line injection technique (Fig. 3d). In these experiments, a small vial containing a freshly prepared suspension of cells, derived from the same SCLC cell line used above, was connected to the venous return line using a T-shaped adaptor, allowing for the slow infusion of a predetermined number of cells using a second peristaltic pump. Similar to the bolus cell line injection experiments described above, slow-injection experiments also resulted in the rapid clearance of cells, represented by the sporadic trajectories of the detected cells (Fig. 3e and Supplementary Fig. 5a). These trajectories indicate that although the tumor cells were introduced continuously over the duration of each experiment, the majority of the cells cleared rapidly from the circulation. Only a small fraction of injected cells was detected over time with much longer time intervals between subsequently detected cells compared to CTC detection rates. In fact, seven-fold fewer cells were detected with the cell line injection experiments compared to endogenous CTCs during the estimated steady-state intervals (*p* < 0.05, Fig. 3f and Supplementary Fig. 5b, c). These results further validate the previous bolus intravenous injection results and again demonstrate the stark differences in the circulatory kinetics between endogenous CTCs and intravenously introduced, cultured cells derived from the same tumors.

Next, we wanted to assess whether the differing half-life times of CTCs spawned by autochthonous tumors and corresponding SCLC cells introduced via the venous circulation were due to differences in cell size, since passage time through a small capillary is strongly size dependent^34–36^. Our group previously demonstrated that measurements of the buoyant mass of cells in suspension can be used as a proxy for their passage time through a microfluidic constriction^37,38^. In order to compare the buoyant mass of CTCs and their model SCLC cell line, we set out to use the sorting functionality^31^ of the CTC counter to sort and enrich a CTC population from a late-stage SCLC-bearing mouse for subsequent buoyant mass measurements using the suspended microchannel resonator (SMR) platform^39,40^. As a control, a viable SCLC cell line population was spiked into a blood sample collected from a healthy mouse (1,000 tdTomato-expressing cells per 100 µL of blood), after which single cells were sorted and enriched using our CTC counter for mass measurements (Supplementary Fig. 6a). Interestingly, both cell populations had a similar buoyant mass distribution (*p* = 0.3, Mann−Whitney−Wilcoxon non-parametric test, Supplementary Fig. 6b), suggesting that the differing cell sizes in these two populations could not be invoked to explain their different persistence times in the circulation. This would suggest, alternatively, that the intrinsic biological properties of these two populations of cells could be the dominant determinants of the unique circulatory kinetics of each population.

We proceeded to examine whether these two cell populations did indeed exhibit distinct biological properties that contributed to their differing lifetimes in the circulation. To this aim, we employed single-cell RNA-Sequencing (scRNA-Seq) to profile the SCLC cell line along with a group of CTCs sorted from a terminal blood sample of an autochthonous SCLC-bearing mouse. Principal Component Analysis (PCA) on highly variable genes revealed stark differences in the two populations, with CTCs associated with a positive PC1 and negative PC2, and the cell line associated with a negative PC1 and positive PC2 (Supplementary Fig. 6c). In order to explore the biological drivers of these differences, we performed correlation analysis between the principal components and expression of Gene Ontology genesets. This analysis revealed that PC1 was positively correlated with genesets for epithelial-to-mesenchymal transition (EMT) and cytoskeletal organization, and was negatively correlated with translation and cell cycle genesets (Supplementary Fig. 6d). This indicates that EMT and cytoskeletal markers are more strongly associated with CTCs, as opposed to the in vitro cell line counterpart, which had higher expression of cell cycle-related genes. These findings also held for PC2, which correlated positively with translation genesets and negatively with cytoskeletal organization and cell stress genesets. Further experiments are needed to validate these findings and explore how these transcriptomic variations influence the behavior of the corresponding cells in circulation, as well as the locations and phenotypes of the distant tumors they form.

### Blood exchange as a method for generating metastases in naïve healthy mice

In two separate experiments, we observed that the direct introduction of as few as 4,000−7,000 SCLC CTCs via blood exchange over a few hours (representing only 1−2% of the average daily shed CTCs) was capable of generating liver and intestinal metastases in healthy recipient mice within two to three months (Supplementary Fig. 7a). Interestingly, the newly developed liver metastatic lesions were also capable of shedding CTCs, as confirmed by the detection of tdTomato-expressing CTCs in the terminal blood of those mice (Supplementary Fig. 7b).

**Fig. 4:**
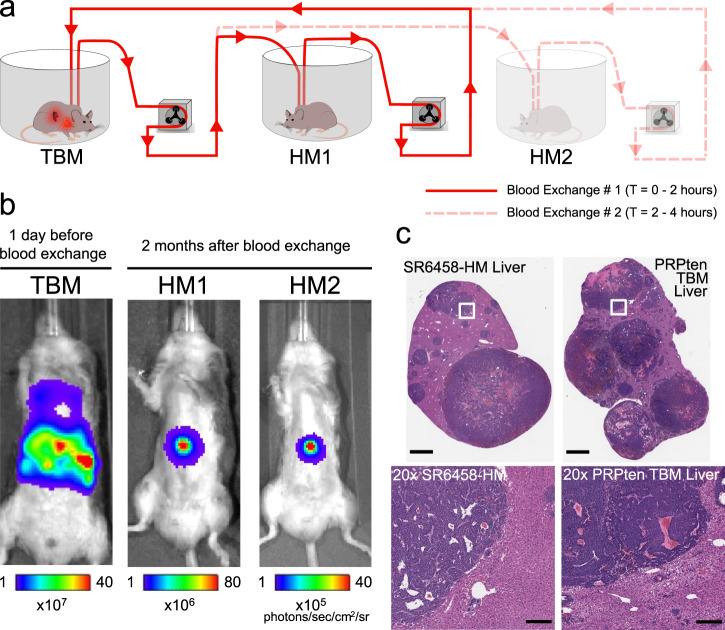
Blood exchange as a method for direct CTC injection for metastasis studies. **a** Schematic demonstrating the serial blood exchange experimental setup in which a single TBM was connected to two healthy counterparts, each for 2 h. **b** Bioluminescent In Vivo Imaging System (IVIS) images demonstrating the tumor burden of the donor (TBM) before the blood exchange and the recipient mice (HMs) when liver metastases were detected two months after blood exchange. **c** Representative H&E-stained liver sections from a blood-exchange recipient mouse (SR6458-HM) and from a tumor bearing mouse (scale bars = 2 mm). Bottom row represents 20× zoomed-in images of the white outlined regions in the top row images (scale bars = 200 µm). These findings were reproduced four times in separate biological replicates. PRPten: Pten^flox/flox^;Rb1^flox/flox^;Trp53^flox/flox^.

We next set out to further investigate the ability of our method to study the metastatic propensity of CTCs introduced into a healthy mouse by blood exchange. Two healthy, immunocompetent mice (IDs: HM1 and HM2) were sequentially connected to a late-stage SCLC GEMM, and approximately 8,000 CTCs were introduced into each healthy mouse over 2 h of blood exchange (Fig. 4a). The healthy recipient mice were longitudinally monitored over several weeks, using in vivo Bioluminescence Imaging (BLI), for tumor development. Both recipient mice developed liver macrometastases approximately two months post blood exchange (Fig. 4b). Interestingly, macrometastases were observed in the liver rather than the lung, even though the CTCs introduced originated from a lung tumor and, having entered via the right jugular vein, encountered the microvessels of the lung in the healthy mouse as the first major impediment to remaining in the circulation. This observation is consistent with the dissemination pattern of the SCLC GEMM as well as clinical findings in which liver metastases are reported as a common dissemination site in SCLC patients^41^. To further validate that the developed tumors in the HMs resemble those of the donor mouse, HM1 was sacrificed following tumor detection for histological analysis. Findings of hematoxylin and eosin (H&E)-stained liver sections from one of the HM lesions confirmed that it displayed histological features of a SCLC metastatic liver tumor (Fig. 4c). Taken together, these findings confirm that the blood exchange technique can be used to create metastatic lesions in healthy recipient mice. Future experiments will be needed to robustly validate these findings, and thoroughly explore the differences between donor and recipient tumors.

## Discussion

Here we demonstrate a blood-exchange method to study the circulatory kinetics of CTCs in mice. The generalizability of our method to different preclinical models was demonstrated by testing it in SCLC, PDAC, and NSCLC murine models, which exhibit vastly different metastatic patterns. Examination of generation rate was last demonstrated using an artificial rat model nearly 45 years ago, when mammary adenocarcinoma CTCs were reported to be shed from solid tumors at a daily rate of several million cells per gram of tissue^42^, which is approximately five times higher than our SCLC estimates (approximately 700,000 cells per gram of SCLC tumor tissue per day), approximately 90 times higher than our PDAC estimates (approximately 40,000 cells per gram of PDAC tumor tissue per day), and approximately 183 times higher than our NSCLC estimates (approximately 20,000 cells per gram of NSCLC tumor tissue per day). Previous estimates of CTC half-life in the mouse circulation have varied from seconds^2,43^ to minutes^13^ to hours^44^, using a variety of detection techniques. In clinical settings, study of CTC half-life has relied on CTC detection in the hours following tumor resection surgery^44^, while preclinical animal models primarily have employed bolus injections of fluorescent tumor cell lines tracked by in-vivo flow cytometry^45,46^. Although longer half-life times of 30−60 min have been previously reported, these measurements were based on only a small fraction of the injected cell population, as the majority of these bulk-injected cells were cleared within seconds^2,3^. We contrast this earlier work with our half-life time estimate of several minutes, obtained from our blood-exchange technique, which involved naturally shed, endogenous CTCs and relied on the positive detection of their real-time entry and exit rates from the circulatory systems of the two connected mice. In contrast to CTCs, when we mimicked these experiments using cell lines in a slow-injection configuration, we observed kinetics that are consistent with previously reported cell line injection experiments, where the majority of the cells are cleared within seconds. Our results raise the question of whether the previous estimates of CTC dwell time in the circulation were strongly influenced by the experimental procedures employed.

The blood-exchange technique discussed here also introduces a method for generating metastases using endogenous CTCs. Traditional methods either use cell lines or require the isolation, ex vivo expansion, and characterization of specific subpopulations of CTCs endowed with metastatic potential, prior to their inoculation into animal models^13,20–22,47^. In our method, CTCs from mice bearing primary and metastatic lesions remain in their original blood and directly enter the circulatory system of the healthy immunocompetent recipient mouse without the need for enrichment or intervening culturing steps that may affect their viability and profoundly change their biological traits. Importantly, our method may extend the time window for studying metastatic disease, particularly in tumor models in which host mice succumb to primary burden before fully-developed metastases can be analyzed^41^. Further studies will be needed to validate the utility of this technique in other cancer types.

In summary, the reported blood-exchange technique and resulting CTC kinetics data can lead to accurate identification of the rate-limiting steps in the blood transport phases of the metastatic cascade. We also envision that our blood-exchange technique can be used to directly and controllably exchange other blood components and study trafficking dynamics of immune cells in various biological contexts within immunology, cancer biology, and aging. Because our blood-exchange technique can be used continuously and longitudinally, it can potentially reveal temporal kinetics that occur on the order of minutes, hours, or days and hence may assist in establishing suitable time windows for maximizing therapeutic efficacy.

## Methods

### The blood-exchange optofluidic platform

The platform is a modified Mouse CTC Sorter^31^ that consists of three major subsystems: a microfluidic device, an optical detection system, and a computer control system. The microfluidic chip is designed with one inlet to a 1 cm-long microfluidic channel (300 µm wide $\times$ 45 µm tall) that bifurcates into two channel outlets (90° apart): one for returning blood to the mouse and the other for collecting the sorted CTC-containing blood sample (Supplementary Fig. 1). The microfluidic device comprises two polymer layers bonded to a glass base layer. The bottom polymer layer implements the pneumatically activated valve structures that control the fluid path through channels in the top layer.

In normal operation, the collection valve is closed and the return valve is open and CTCs are just enumerated as they pass through the system. If a CTC is to be collected, the return valve is closed, and the collection valve opens momentarily to deflect a small amount of blood containing the CTC. At a flow rate of 30 µL/min, the average sort volume is 127 ± 47 nanoliters/CTC. Minimal cell losses (of less than 3%) within the CTC Counter (including the microfluidic chip, the peristaltic pump, and the connecting tubing) were verified by flowing a predefined number of tdTomato-expressing tumor cells through both systems sequentially and tracking the number of cells detected on each pass (Supplementary Fig. 2b).

### Device fabrication

The fabrication of the microfluidic chip for the CTC counter starts with standard soft lithographic techniques on two four-inch silicon wafers^31^. A single layer of photoresist (SU8 2050, Microchem, Newton, MA) is patterned to create the pneumatic channels on the valve actuation wafer. For the blood flow channel, AZ9260 positive resist is exposed, developed, and then reflowed at 120 °C for 10 min to create the rounded channel profile necessary for a complete valve seal. Once the master molds are fabricated for both the actuation and flow channel layers, a mixture of PDMS (Polydimethylsiloxane) and its curing agent (SYLGARD 184 A/B, Dowcorning, Midland, MI, USA) at a 10:1 ratio was spun on top of the actuation wafer to a thickness of 50 µm and baked in an oven set to 65 °C for 3 h. For the flow channel layer, the mixture was poured to a thickness of ~1 cm and cured at 65 °C for 3 h. Afterwards, the flow channel layer was peeled off and punched with a 0.75 mm puncher (Harris Uni-Core, Ted Pella Inc., Reading, CA) to define the inlet and outlets to and from the flow channel, respectively, and diced to prepare for bonding. The flow channel devices and the actuation layers were then treated with oxygen plasma (100 watt, 1 ccm, 140 torr, 10 s). Next, the flow layer was aligned to the actuation layer and baked in an oven at 60 °C. After 15 min, the assembled PDMS layers were peeled off the wafer and punched with a 0.75 mm puncher to define inlets to the actuation channels. The assembled PDMS layers were treated with oxygen plasma (100 watt, 1 ccm, 140 torr, 10 s) for irreversible bonding to a glass slide (Fisherbrand 1×3”, Fisher Scientific, Pittsburgh, PA). Prior to flow experiments, the device was aligned within the system to project the two laser lines across the flow channel 8 mm away from the valve actuation region. The device was then primed with Heparinized-Saline (diluted to 100 USP units per mL, NDC 25021-400-30) to prevent clotting within the microfluidic channel.

### CTC counter optical setup

The CTC Counter consists of two optical trains aligned vertically. The top optical train consists of a dichroic filter to reflect the excitation beam (532 nm) onto the sample and transmit the emitted signal (greater than 532 nm). The second dichroic mirror and longpass filters, placed such that they are constantly directly above the detection region during normal operation, pass a filtered fluorescence signal to the PMT (Hamamatsu H10722-20, spectral response: 230−920 nm, peak sensitivity wavelength = 630 nm) by further blocking the 532 nm laser lines with a notch filter. A 90:10 (T:R) beam splitter is added before the PMT to allow for imaging of the detection region for device alignment purposes during initial experimental set up. Determination of the optimal excitation and emission focal planes across the center of the microfluidic channel in the detection region is based on on-chip alignment marks placed near the detection area viewed by a dedicated PMT camera (top camera in Supplementary Fig. 3b). The bottom optical train, for verifying proper microvalve functionality and sorting mechanism of the chip, consists of similar components to the top optical train but shifted laterally by approximately 1 cm towards the outlet region.

### PMT data processing

The PMT module generates an output voltage (which is more resistant to external noise than when extracting current) that is sampled by a NI USB-6211 16-bit multifunctional DAQ analog-to-digital converter. Sampling of the raw PMT data is done at 30,000 data points per second. The original unfiltered raw PMT data (gray in Fig. 1b) is unstable and fluctuates with the pulsatile blood flow caused by the peristaltic pump. Therefore, a median filter of rank 100 (in other words, 100 elements are used to compute the median filter to the left side of the current data point) is subtracted to remove low frequency drift or any DC offset. Afterwards, the median-filtered data is passed through LabVIEW’s built-in lowpass Chebyshev filter (blue or orange traces in Fig. 1b). Chebyshev filters were chosen for smoothing the raw PMT data because they are known to minimize peak error in the passband, monotonically decrease magnitude response in the stopband, and have a much sharper roll-off than Butterworth filters. Detection threshold (horizontal line in Fig. 1b insets) is set to at least five times the standard deviation of the shot noise.

Real-time recorded PMT data are then divided into segments and analyzed in MATLAB against a set of peak parameters to identify real CTC peaks. Parameters such as peak maximum height, first to second peak height ratio, maximum and minimum peak separation in data points are all determined based on characterization experiments using tdTomato-expressing CTCs and cell lines (see supplementary section of ref. 28). The transfer rates *r*_1_ and *r*_2_ were used for the calculation of half-life and generation rate in Microsoft Excel. Figures were plotted in MATLAB and GraphPad Prism.

### Mice

All animal-based procedures were approved by the Massachusetts Institute of Technology Committee on Animal Care (CAC), Division of Comparative Medicine (DCM). Animals were housed on hardwood chip bedding, with a 12/12 light dark cycle, with a temp of 70 °F +/−2 and a humidity range of 30−70%. All SCLC mice were maintained on a mixed C57BL/6;129/Sv background. Rosa26^LSL-tdTomato/LSL-tdTomato^ mice were obtained from Jackson Laboratories (Gt(ROSA)^26Sortm14(CAG-tdTomato)Hze^) and crossed into the PRP-L/L model to obtain Trp53^fl/fl^; Rb1^fl/fl^; Pten^fl/fl^; Rosa26^LSL-tdTomato/LSL-Luciferase^ mice^26^. Tumors in TBMs are initiated by delivering 2 × 10^8^ plaque forming units (pfu) of adenovirus expressing Cre recombinase under the control of a CGRP promoter (Ad5-CGRP-Cre) into the trachea^28^. Adenoviral stocks were purchased from the Viral Vector Core Facility at the University of Iowa Carver College of Medicine.

For PDAC experiments, all animals, including KPT mice (Kras^LSL-G12D/+^; Trp53^fl/fl^; Rosa26^LSL-tdTomato/LSL-tdTomato^), were maintained on a C57BL/6 background. In order to derive PDAC organoids, the normal pancreas of KPT mice was minced and dissociated in pancreas digestion buffer [1× PBS, 125 U/mL collagenase IV (Worthington)] for 20 min at 37 °C. Cells were plated in Matrigel^48^, followed by ex vivo transformation, via delivery of Ad-CMV-Cre (University of Iowa Viral Vector Core). Orthotopic transplantation of organoids was performed with minor modifications to previously reported protocols for orthotopic transplantation of pancreatic monolayer cell lines^49^. A small incision was made in the left subcostal area and the spleen and pancreas were exteriorized using ring forceps. A 30-gauge needle was inserted into the pancreatic parenchyma parallel to the main pancreatic artery and 100 μL (containing 1.25 × 10^5^ organoid single cells in 50% PBS + 50% Matrigel) was injected into the pancreatic parenchyma. Successful injection was visualized by formation of a fluid-filled region within the pancreatic parenchyma without leakage. The pancreas and spleen were gently internalized and the peritoneal and skin layers were sutured independently using 5-0 vicryl sutures. For orthotopic transplantation, syngeneic C57BL/6 J mice (aged 6−12 weeks) were transplanted. Male pancreatic organoids were only transplanted back into male recipients. Two genetically-defined pancreatic organoid lines were used to initiate tumors: an ex vivo transformed (KPT) line and a recently described ex vivo transformed organoid line harboring a defined neoantigen (KP;mScarletSIIN)^50^.

Autochthonous PDAC tumors were also initiated in KPT mice by retrograde pancreatic duct delivery of Ad-Ptf1a-Cre virus (7 × 10^8^ pfu/mouse; University of Iowa Viral Vector Core)^27,50^. A small incision was made in the anterior abdomen (approximately 2−3 cm), the intestines and liver were gently repositioned until the biliary tree was well visualized. A microclip was placed over the common bile duct to prevent flow of the viral particles into the liver or gallbladder, forcing the viral vector retrograde through the pancreatic duct. The common bile duct was then cannulated with a 30-gauge needle at the level of the sphincter of Oddi and 150 μL of virus was injected over the course of 30 s.

To initiate NSCLC tumors, Kras^LSL-G12D/+^; Trp53^fl/fl^; Rosa26^LSL-tdTomato/LSL-Cas9^ mice^28–30^ were infected with adenovirus expressing Cre recombinase under the control of the alveolar type II cell-specific promoter, Sftpc (Ad5mSPC-Cre; University of Iowa Viral Vector Core). 1 × 10^8^ plaque-forming units (pfu) were delivered by intratracheal instillation (i.t.) at the start of the experiment.

### Cannulation surgery

All animal-based procedures were approved by the Massachusetts Institute of Technology Committee on Animal Care (CAC), Division of Comparative Medicine (DCM). Candidate mice for the arteriovenous shunt surgery were identified by either in vivo bioluminescence imaging using the IVIS Spectrum In Vivo Imaging System (PerkinElmer), ultrasound using the Vevo 3100 LAZR-X (FUJIFILM-Visualsonics), or microCT using the SkyScan 1276 (Bruker). Catheters are inserted into the right jugular vein and the left carotid artery and are externalized using standard cannulation surgical techniques in anesthetized mice^31^.

### Imaging of CTCs

Purified CTCs were stained with 1:100 anti-mouse CD45-FITC (Invitrogen Cat #11-0451-82) for 20 min and 1:100 DAPI staining solution (Miltenyi Cat #130-111-570) before imaging on a Nikon A1R Ultra-Fast Spectral Scanning Confocal Microscope using NIS Elements.

### Calculation of tumor masses

Lung tumor mass was calculated by subtracting the average mass of healthy (uninfected) PRPten mouse lungs from the mass of the resected lungs (including the tumor). We found the approximate mass of the lung tumor compartment to be in the range of 450−650 mg at this late stage for SCLC (five to six months post tumor initiation) and around 150−500 mg at this late stage of NSCLC (four months post tumor initiation). PDAC tumor volumes were estimated using Vevo LAB software (FUJIFILM Visualsonics, Inc.) to analyze ultrasound imaging. Tumor masses were not measured directly. Instead, tumor masses were estimated from a derived tumor volume to tumor mass formula, which was generated by fitting a linear line (with *R*^2^ = 0.84) to previously recorded internal tumor volume and weight values for similar tumors from mice of comparable age and tumor burden to the mice used in this study:

6 $${{{\rm{tumor}}}}\,\,{{{\rm{weight}}}} = {1.912}\, \times {{{\rm{tumor}}}}\,\,{{{\rm{volume}}}} - {50.09} \,\,[{{\rm{mg}}}]$$

Tumor mass is in milligrams and tumor volume is in cubic millimeters. Total tumor cell numbers were calculated using the assumption that 1 g of primary tumor contains approximately 10^9^ tumor cells^51^, and that this tumor serves as the main source of shed CTCs.

### Cell line injection studies

A murine SCLC cell line (AF3291LN) isolated from SCLC tumors of Trp53^fl/fl^; Rb1^fl/fl^; Pten^fl/fl^; Rosa26^LSL-tdTomato/LSL-Luciferase^ mice was used^33^. For bolus cell line injections, a population of at least 1 × 10^6^ cells was harvested from a flask, rinsed with saline, and centrifuged at 300 g for 5 min. Pelleted cells were then re-suspended in saline and counted using the CTC counter to dilute the sample to a dosing concentration of 25,000 cells per 40 µL.

For slow injection experiments, similar washing steps were executed, but the final cell density was reduced to a desired concentration between 3,000 and 20,000 cells/mL. The cell-suspension vial was connected to a second peristaltic pump that infused the cells intravenously through a T-adaptor at a fixed flow rate of approximately 2 µL/min. Throughout the injection experiment, the cell suspension vial was replaced with a new, well-mixed cell-suspension vial every 15 min to ensure a consistent infusion of viable cells. To determine the approximate injection count over time (blue trajectories in Fig. 3e and Supplementary Fig. 5a), a small sample from each freshly-prepared vial was taken and counted on a separate CTC counter.

### SMR experiment

The silicon-based SMR chip is the core component of the SMR system and consists of a sealed microfluidic channel that runs through the interior of a cantilever resonator^40^. As a cell in suspension flows through the cantilever, it transiently changes the cantilever’s resonant frequency in proportion to its mass. A fluorescent readout was integrated into the SMR device to detect the tdTomato-expressing CTCs from a heterogenous sample. In this modified SMR system, an excitation laser beam is focused into a 500 µm line and aligned across the inlet bypass channel of the SMR chip (Supplementary Fig. 6a). Emitted signal from a tdTomato-expressing CTC passing under the laser line is detected by a PMT (similar to the real-time CTC counter) to induce an automated fluidic direction change that slowly loads the cell into the cantilever section for mass measurements. Prior to their mass measurement, both samples (CTCs and enriched cell line) are re-suspended in growth media (DMEM with 10% FBS).

### CTC enrichment for single-cell analyses

CTC-enriched blood sorted from the CTC counter is further purified through sequential dilution. The pooled CTCs (in approximately 100 nL/CTC) are diluted to 500 µL in cell culture media (DMEM + 10% FBS). The diluted blood is run through the CTC counter again, and fluorescent CTCs are each re-sorted into approximately 100 nL of media and re-diluted. After three dilution steps in media, the purified CTCs are diluted a final time to 500 µL in RNase-free PBS, run through the CTC counter, and each sorted CTC is collected into a PCR tube containing 7 µL of 2 × TCL lysis buffer (Qiagen) with 2% v/v 2-mercaptoethanol (Sigma). The samples are immediately frozen on dry ice and subsequently stored at −80 °C until library preparation and sequencing. The complete enrichment process, from whole blood to individually sorted CTCs for single-cell analyses, results in a typical dilution between 3.9 × 10^5^ and 6.3 × 10^6^, depending on the number of detected CTCs.

### Single-cell RNA-sequencing sample preparation

CTC and primary tumor samples in TCL supplemented with 1% (v/v) 2-mercaptoethanol buffer were processed through Smart-Seq2^31,52^. Cellular nucleic acids from each lysed single cell were extracted from TCL lysis buffer using a 2.2× (v/v) RNA SPRI (RNA-clean AMPure beads, Beckman-Coulter). After, we performed reverse transcription with Maxima enzyme (Thermo scientific), and then PCR using KAPA Hotstart Readymix 2× kit (KAPA biosystems) (primers can be found in Supplementary Table 4). Following quantification and quality control analysis by Qubit DNA quantification (Thermoscientific) and tape station (Agilent), the post-PCR whole transcriptome amplification (WTA) products from each single cell were transformed into sequencing libraries using a Nextera XT kit (Illumina) and unique 8 bp DNA barcodes. cDNA libraries were pooled, quantified, and sequenced on an Illumina NextSeq 500 to an average depth of 1.2 × 10^6^ reads/cell.

### Analysis of raw sequencing data

Following sequencing, BCL files were converted into merged, demultiplexed FASTQs. Paired-end reads were mapped to mm10 mouse transcriptome (UCSC) with Bowtie 2. Expression levels of genes were log-transformed transcript-per-million (TPM[i,j]) for gene i in sample j, estimated by RSEM in paired-end mode. For each cell, we enumerated genes for which at least one read was mapped, and the average expression level of a curated list of housekeeping genes. We excluded from analysis profiles with fewer than 500 detected genes or below 375,000 total reads, though downstream results were consistent with more and less stringent cutoffs. Principal component analysis (PCA) was performed on all variable genes except Gm, RP, and Hb genes as initial results indicated a dominant method of sorting signature within the dataset driven by these genes.

### PC correlation analysis

To understand correlative effects of metastasis-related genesets on the PC separation of CTCs vs cell line (Supplementary Fig. 6c), module scores were added for each cell for several Gene Ontology genesets [Epithelial to Mesenchymal Transition (GO:0001837), Cytoskeleton Organization (GO:0007010), Regulation of Cell Stress (GO:0080135), Fluid Shear Stress (GO:0034405), Cell Cycle (GO:0007049), Cytoplasmic Translation (GO:0002181), Translation Initiation (GO:0006413)] using the AddModuleScore function in Seurat 3.0. Then, Pearson Correlation coefficients were calculated between the module scores for each geneset and both PCs 1 & 2. Finally, the resulting *R* values were visualized using the Complexheatmap package (Supplementary Fig. 6d), and *p* values were calculated using *R* value and number of samples. To confirm that PC separation was not driven by quality variations, cutoffs were modulated to ensure comparable levels of quality control metrics. The same trends in PC correlation to GO genesets was found, and quality control metrics had no significant correlation.

### Reporting summary

Further information on research design is available in the Nature Research Reporting Summary linked to this article.

## Supplementary information

Supplementary Information

Reporting Summary

## Data Availability

The single-cell RNA sequencing data reported in this paper is deposited in the NCBI Sequence Read Archive, accession Number PRJNA670615. The remaining data are available in the Article or Supplementary Information.

## Code availability

CTC processing code can be found at https://github.com/amiller92/CTCPeakAnalysis.
