# Supplementary material for: Measuring kinetics and metastatic propensity of CTCs by blood exchange between mice
Source: Nat Commun. 2021 Sep 28;12:5680. doi: 10.1038/s41467-021-25917-5 (PMC8479082; doi:10.1038/s41467-021-25917-5)
Supplement: Supplementary file 1 — Supplementary Information [file 41467_2021_25917_MOESM1_ESM.pdf]

SUPPLEMENTARY INFORMATION

Measuring kinetics and metastatic propensity of CTCs by blood exchange between mice

Bashar Hamza[1,2*], Alex B. Miller[2,3*], Lara Meier[2,4,5,], Max Stockslager[2,6], Sheng Rong Ng[2,7], Emily M. King[2], Lin Lin[2], Kelsey L. DeGouveia[2,8], Nolawit Mulugeta[2], Nicholas L. Calistri[2], Haley Strouf[2], Christina Bray[2], Felicia Rodriguez[2,9], William A. Freed-Pastor[2,10], Christopher R. Chin[2], Grissel C. Jaramillo[2,7], Megan L. Burger[2], Robert A. Weinberg[2,7,11], Alex K. Shalek[3,12,13,14,15,16], Tyler Jacks[2,7], and Scott Manalis[2,6,9,14,17†]

[1]Department of Electrical Engineering and Computer Science, Massachusetts Institute of Technology, Cambridge, MA 02139, USA.
[2]David H. Koch Institute for Integrative Cancer Research, Massachusetts Institute of Technology, Cambridge, MA 02142, USA.
[3]Harvard-MIT Department of Health Sciences and Technology, Institute for Medical Engineering and Science, Massachusetts Institute of Technology, Boston, MA 02142, USA.
[4]Department of Oncology, Hematology and Bone Marrow Transplantation with Section Pneumology, Hubertus Wald Comprehensive Cancer Center Hamburg, University Medical Center Hamburg-Eppendorf, Martinistrasse 52, 20246, Hamburg, Germany.
[5]Department of Tumor Biology, Center of Experimental Medicine, University Medical Center Hamburg-Eppendorf, Hamburg, Germany.
[6]Department of Mechanical Engineering, Massachusetts Institute of Technology, Cambridge, MA 02139.
[7]Department of Biology, Massachusetts Institute of Technology, Cambridge, MA 02139.
[8]Department of Biomedical Engineering, Wentworth Institute of Technology, Boston, MA 02115.
[9]Department of Biological Engineering, Massachusetts Institute of Technology, Cambridge, MA 02139.
[10]Department of Medical Oncology, Dana-Farber Cancer Institute, Boston, MA 02215.
[11]Whitehead Institute for Biomedical Research, Cambridge, MA 02142.
[12]Department of Chemistry, Massachusetts Institute of Technology, Cambridge, MA 02139.
[13]Institute for Medical Engineering and Science, Massachusetts Institute of Technology, Cambridge, MA 02139.
[14]Broad Institute of MIT and Harvard, Cambridge, MA 02142.
[15]Ragon Institute of MGH, MIT and Harvard University, Cambridge, MA 02139.
[16]Department of Immunology, Massachusetts General Hospital, Boston, MA 02114.
[17]Ludwig Center at MIT's Koch Institute for Integrative Cancer Research, Cambridge, MA 02142.
*These authors contributed equally
†corresponding author (srm@mit.edu)

Supplementary Figures 1-7
Supplementary Tables 1-4

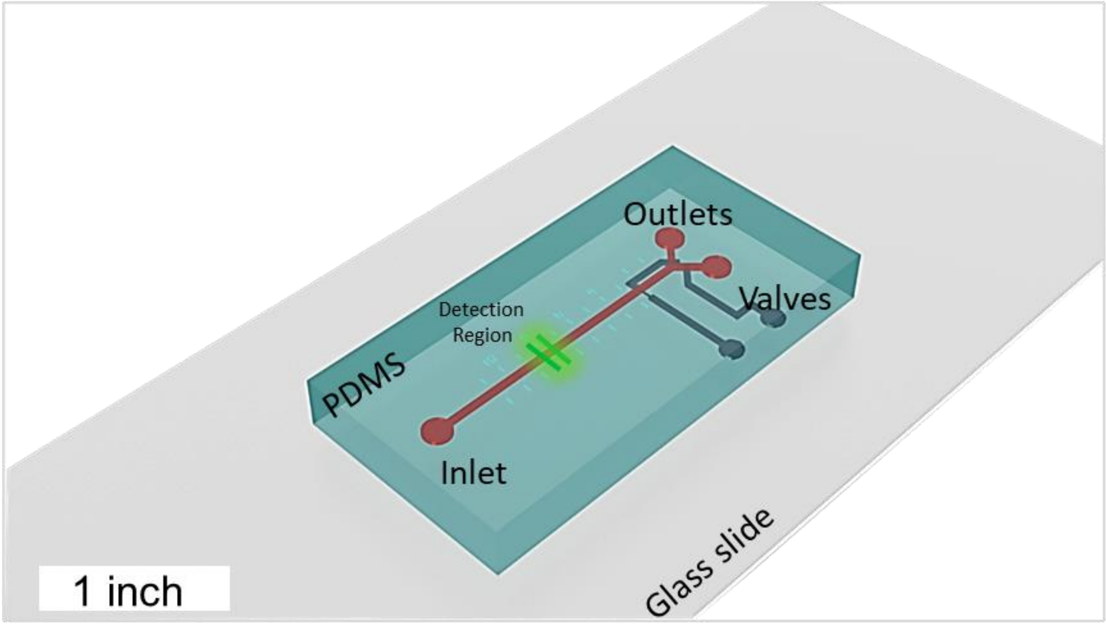

Supplementary Fig. 1 | Three-dimensional rendering of the PDMS-based CTC counter microfluidic device for counting or sorting CTCs in real-time. CTCs pass through dual excitation laser lines focused near the inlet of the device (Detection Region). The device has two outlets, one for returning the blood to the mouse and the other for collecting CTCs, when the sorting functionality is activated. Microvalves near the outlet control blood flow out of the microfluidic chip.

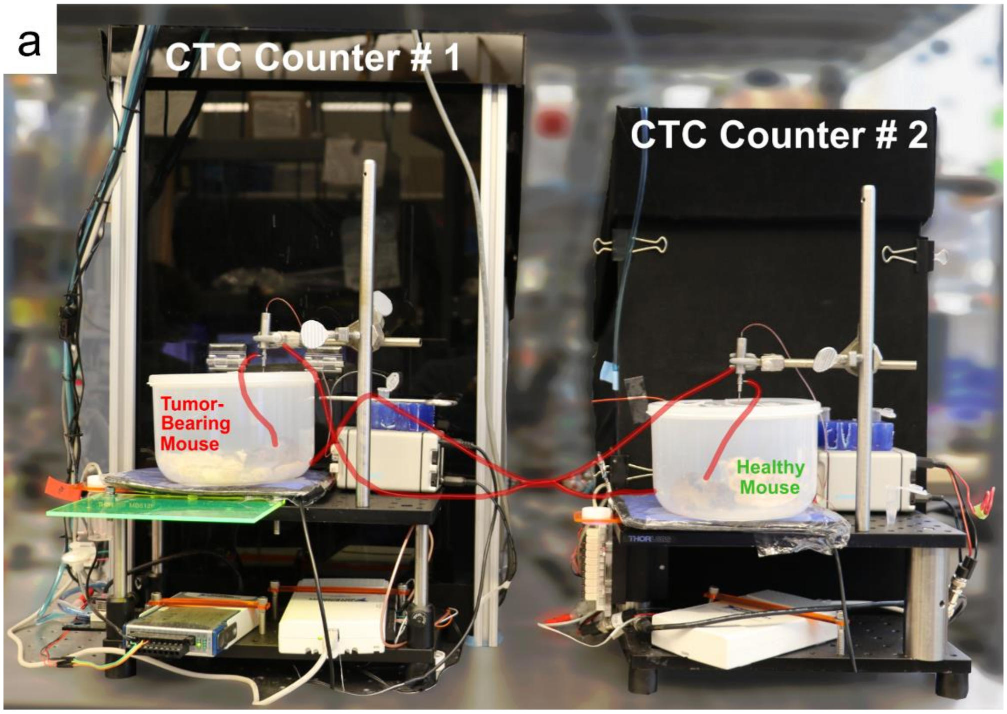

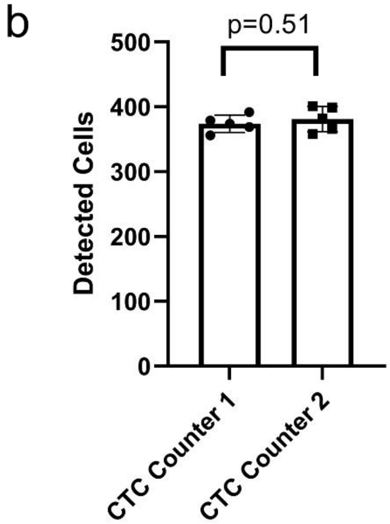

Supplementary Fig. 2 | Blood exchange setup and validation. (**a**) A front view demonstrating the blood-exchange set up. Two mice (TBM and HM) with tethered externalized carotid artery and jugular vein catheters move freely in a small container. Peristaltic pumps withdraw blood from the carotid artery of each mouse into the inlets of the optofluidic CTC counters (contained within the black boxes behind each mouse container). Return blood catheters (highlighted in red) transport blood back to the jugular vein of the other mouse to complete the blood flow loop without loss. Both systems are controlled by two separate LabVIEW controllers and the entire system is suspended on an optical table to isolate vibrational noise. (**b**) Minimal losses of CTCs within the CTC Counters and the tubing were validated by flowing approximately 400 SCLC cells suspended in 50 µL of PBS in a loop between CTC Counters 1 and 2 for five repetitions. Data is shown as the mean and standard deviation of the detected cell counts on each counter, one from each cycle per counter, for a total of five cell counts per counter (2-tailed t test; $p=0.51$).

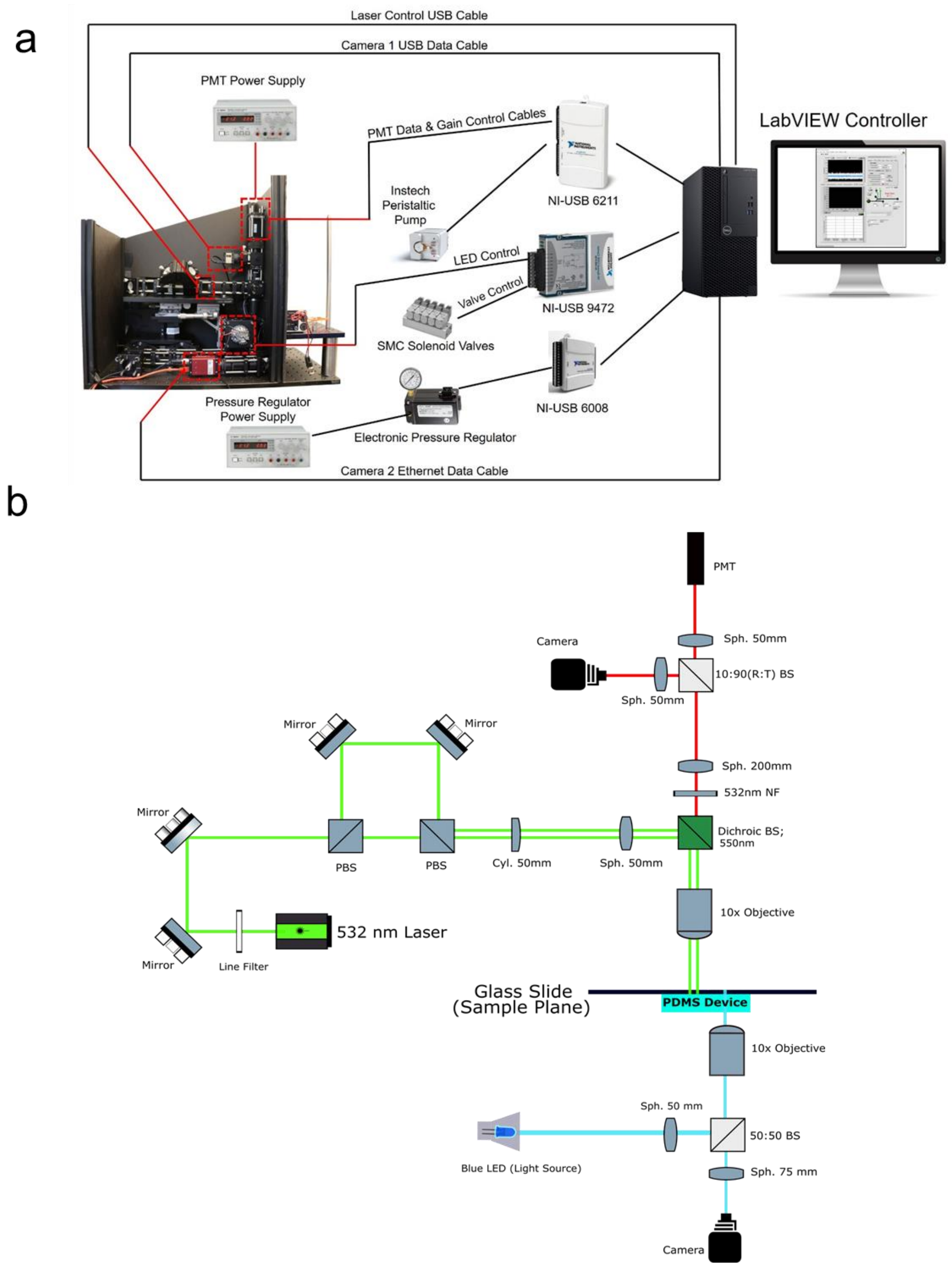

Supplementary Fig. 3 | Schematics of the electrical and optical components of CTC sorter. The main hardware and optical components of the CTC counters. (**a**) Side-view image of one of the CTC counters with visual illustrations of the different interfacing components for data flow. (**b**) Schematic of the optical components of the CTC counters. A single excitation source (532 nm Laser) on the top optical train is filtered, split, and then projected on the sample plane for CTC detection within the PDMS-based microfluidic device. Emitted signal is filtered prior to being focused on a PMT for signal detection. The bottom optical train is used for real time monitoring of blood flow at the outlet region (using a camera).

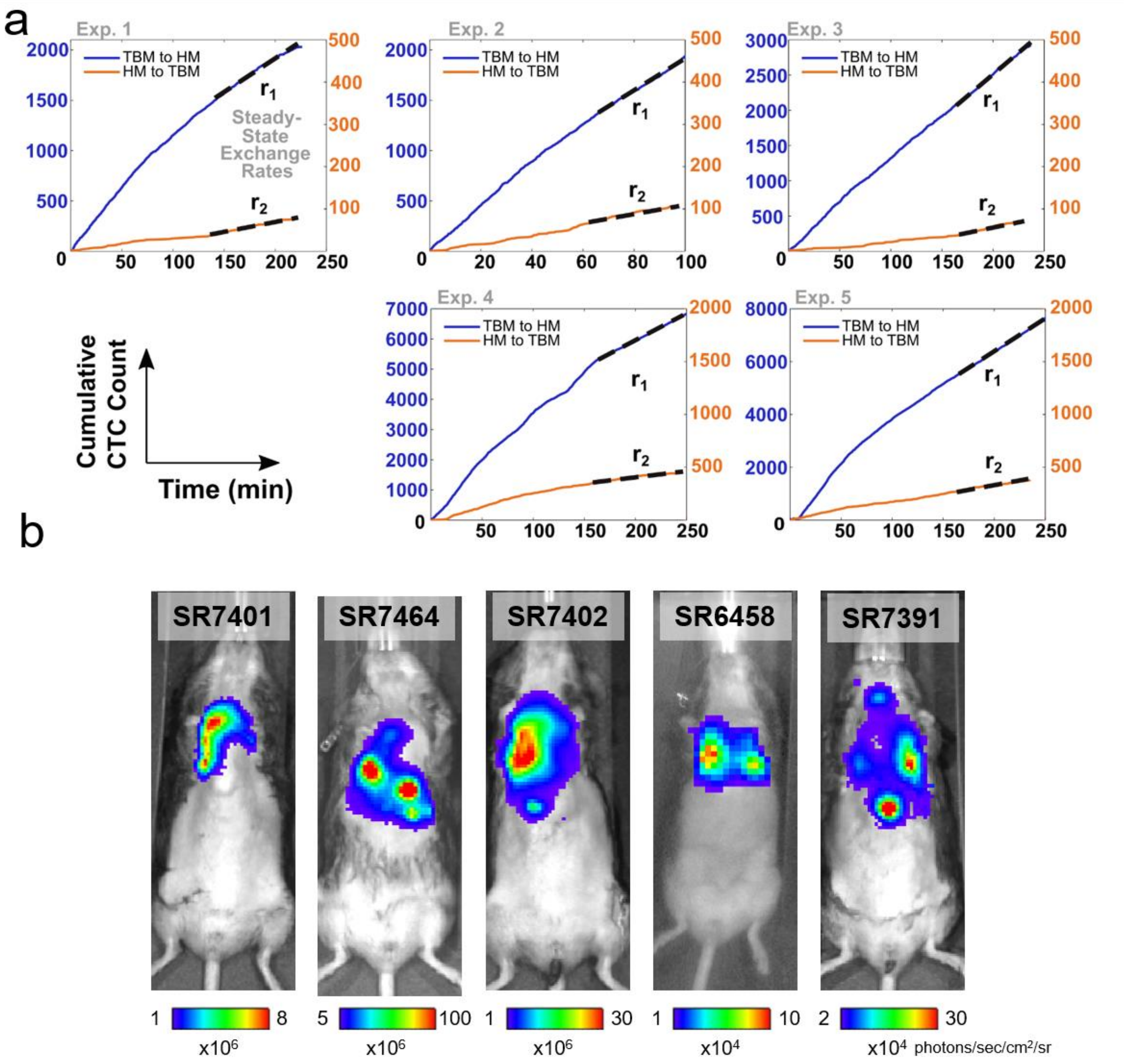

Supplementary Fig. 4 | CTC exchange rates in late-stage SCLC mice. (**a**) Five cumulative CTC count charts over time representing the counted CTCs when exchanged between five different pairs of mice (five late-stage SCLC TBMs and five HMs). In each experiment a TBM is connected to a HM of the same sex, age, and genotype. Dotted lines represent the best fit lines, which were used to extract the steady-state exchange rates between the two mice (r_1 and r_2). (**b**) Bioluminescence IVIS images of each of the TBMs demonstrating a visible tumor burden in the days or weeks prior to their blood-exchange experiments with healthy counterparts.

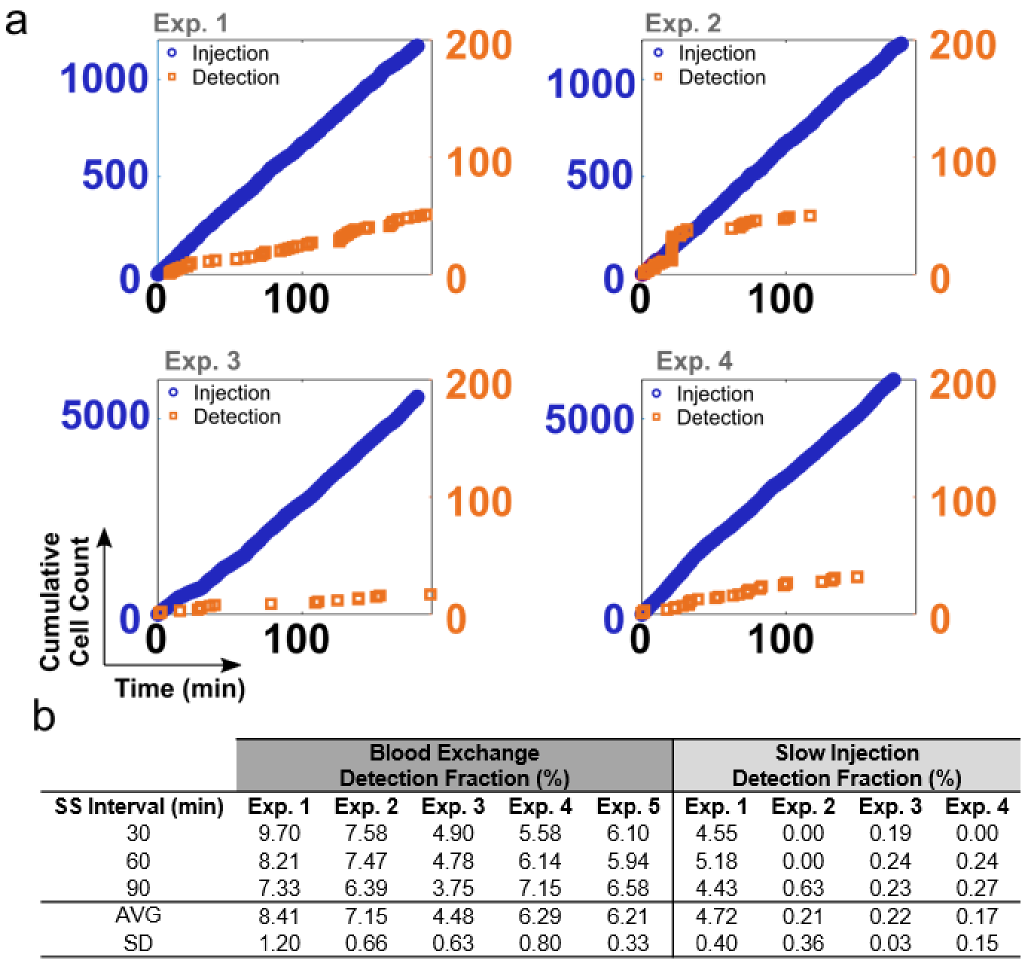

b

	Blood Exchange Detection Fraction (%)					Slow Injection Detection Fraction (%)			
SS Interval (min)	Exp. 1	Exp. 2	Exp. 3	Exp. 4	Exp. 5	Exp. 1	Exp. 2	Exp. 3	Exp. 4
30	9.70	7.58	4.90	5.58	6.10	4.55	0.00	0.19	0.00
60	8.21	7.47	4.78	6.14	5.94	5.18	0.00	0.24	0.24
90	7.33	6.39	3.75	7.15	6.58	4.43	0.63	0.23	0.27
AVG	8.41	7.15	4.48	6.29	6.21	4.72	0.21	0.22	0.17
SD	1.20	0.66	0.63	0.80	0.33	0.40	0.36	0.03	0.15

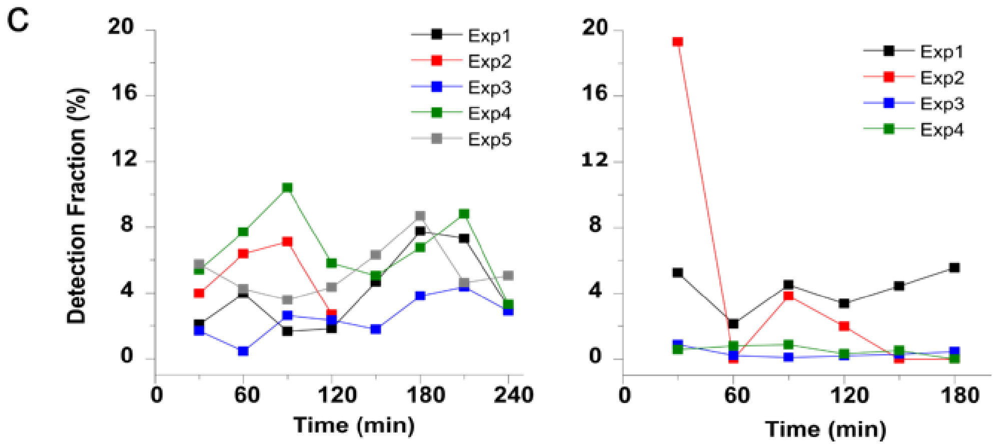

Supplementary Fig. 5 | Detection fraction comparison of slow injection and blood exchange. (**a**) Cumulative injected (blue) and detected (orange) cell counts from four slow-injection experiments. Fluorescent SCLC cells were suspended in saline at pre-determined concentrations and then slowly introduced into the circulatory system of four different healthy recipient mice. Cell counts in blood were monitored for three hours during the slow injection (orange). (**b**) Table summarizing the fraction of detected cells to total injected cells ("Detection Fraction") in the steady-state intervals at the end of each of the nine experiments (five blood exchange and four slow-injection experiments). (**c**) Detection fraction over 30-minute intervals throughout the blood-exchange (left) and slow-injection (right) experiments.

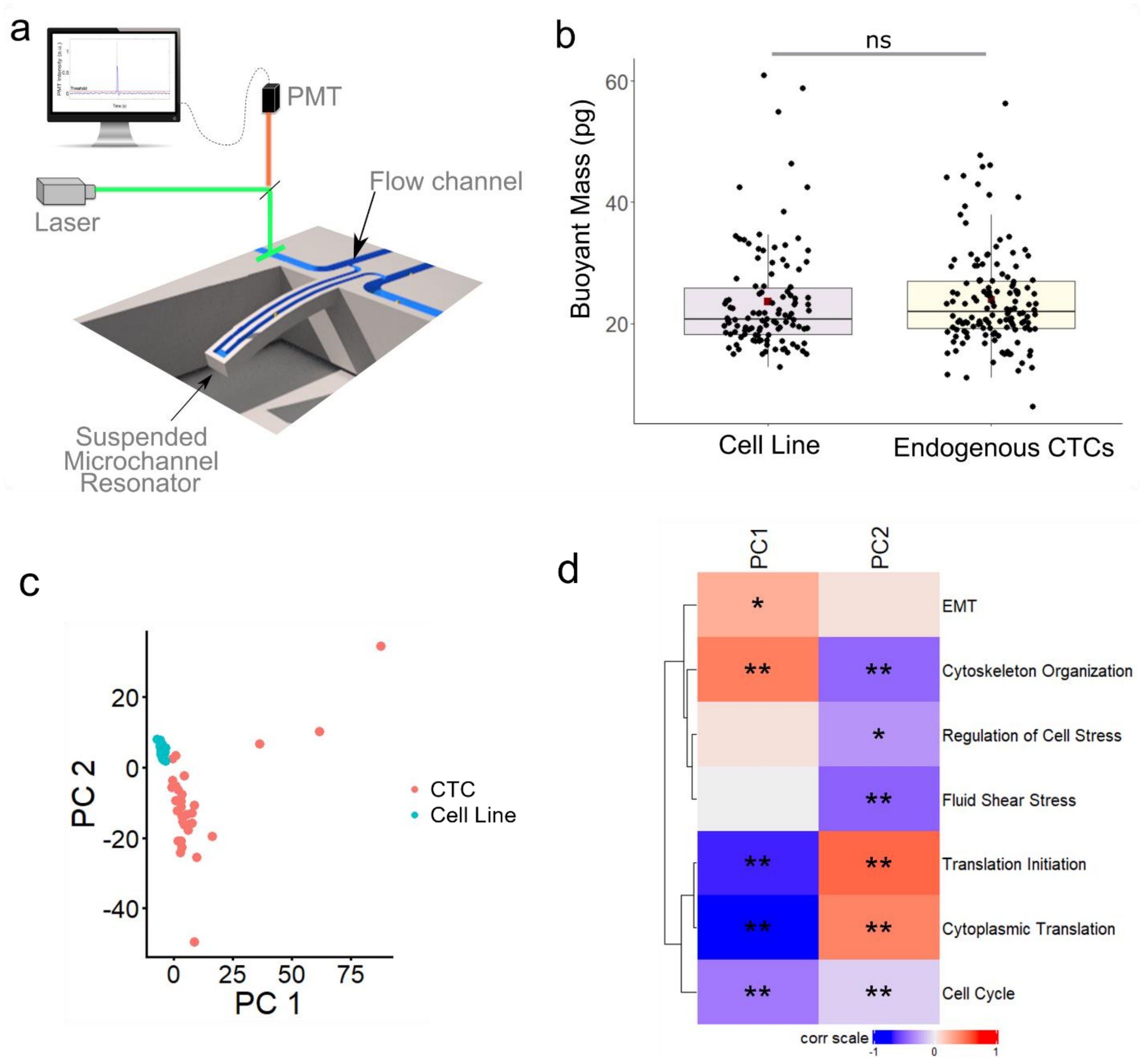

Supplementary Fig. 6 | Comparison of mass and gene signature of endogenous CTCs to intravenously injected SCLC cell line. (**a**) Schematic demonstrating the main components of the suspended microchannel resonator (SMR) platform that was designed and built for measuring the buoyant mass of single fluorescent cells in heterogeneous samples. A laser excitation source is aligned across a bypass channel upstream of the mass sensor (cantilever). Upon the detection of a target fluorescent cell or CTC, an automated active-loading system is triggered to allow for the flow to be directed through the cantilever in order to measure the cell's buoyant mass. (**b**) Box plot demonstrating the buoyant mass distribution of detected CTCs (N=140 cells) and the SCLC cell line (N=114 cells). Box plot lower and upper hinges correspond to the first and third quartiles, center line represents median value, red square represents mean value, lower whisker extends to the smallest observation above 1.5×inter-quartile range, upper whisker extends to the largest observation below 1.5×inter-quartile range). No significant difference was observed between the two groups (ns: not significant; $p = 0.3$, two-sided Mann-Whitney-Wilcoxon non-parametric test). (**c**) Principal Component Analysis (PCA) plot showing separation of scRNA-Seq data between true CTCs and the cell line population. (**d**) Heatmap showing correlation coefficients between the PCs 1 & 2 with expression of select Gene Ontology genesets. Color represents Pearson Coefficient, R. *$p<.01$; **$p<.0001$.

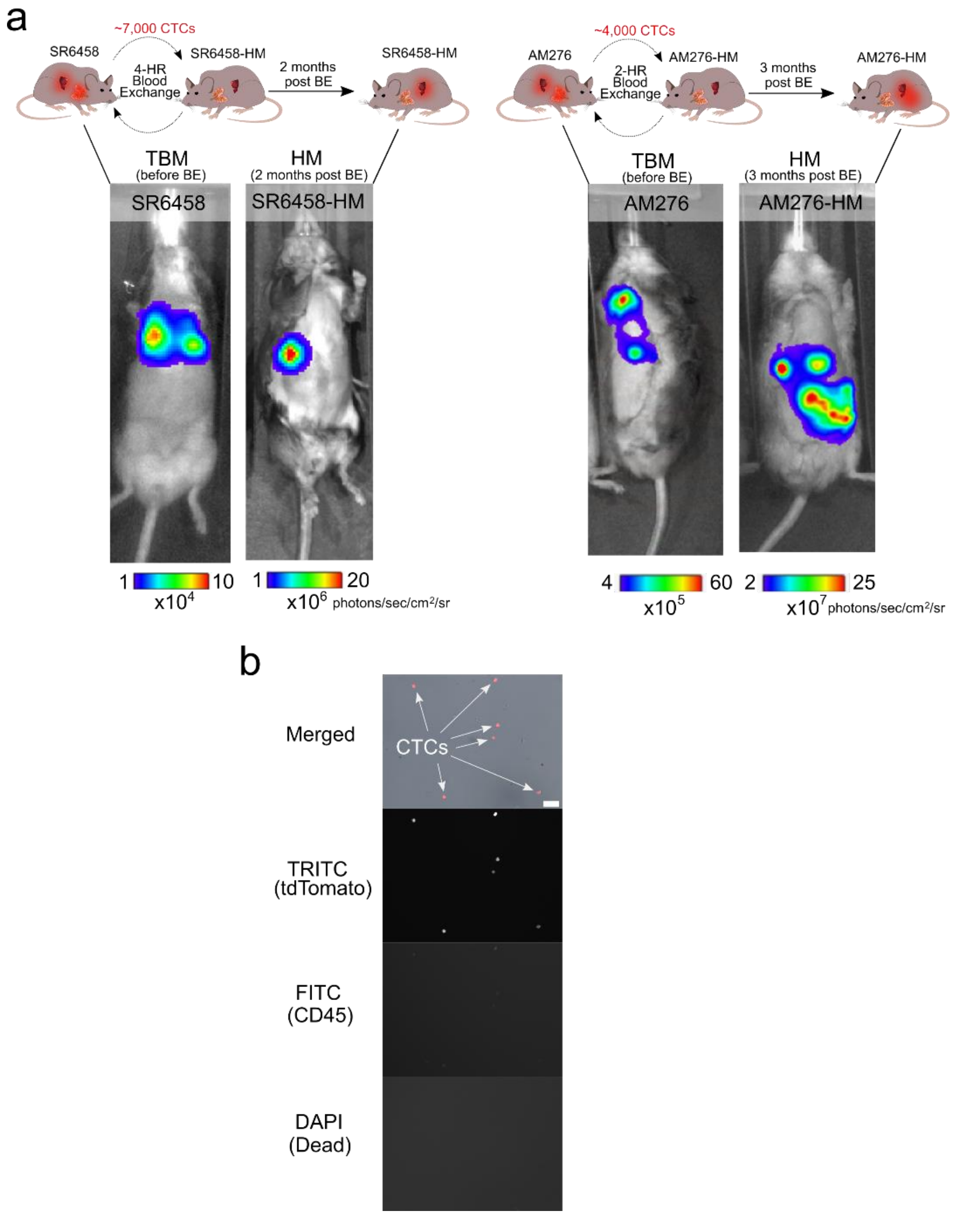

Supplementary Fig. 7 | Imaging of blood-exchange induced metastatic tumors and CTCs in recipient mice. (**a**) Bioluminescence IVIS images of two SCLC TBMs prior to blood exchange experiments and two originally healthy counterparts two months after the blood-exchange experiments. Superimposed rainbow-colored regions represent the detectable lung and metastatic liver tumor burdens in the TBMs and the liver/intestinal burdens in the HMs. (**b**) 10x microscopy images of different fluorescent channels showing CTCs single-cell sorted into wells of a glass-bottom multi-well plate from the terminal blood of the HM two months after the blood-exchange experiment (scale bar = 20 µm). This finding was reproduced four times in separate biological replicates.

Supplementary Table 1 | Real-time and terminal blood concentrations of CTCs. Comparison between CTC concentrations as measured through real-time scans in the live animal using the CTC counter and terminal blood scans collected after the real-time scan from the same animal.

	CTCs/mL	
Exp #	Real-Time Scan	Terminal Blood
1	400	318
2	893	505
3	287	296
4	555	458

Supplementary Table 2 | Raw data of blood exchange experiments. Table summarizing the main parameters extracted from the CTC counts during the blood exchange experiments. Error (“Err”) represents the propagated error due to the absolute uncertainty (s.d.) in the r_1 and r_2 estimates.

		TBM ID	Exp. #	Q (mL/min)	V (mL)	r_1 (cells/min)	r_1 SD	r_2 (cells/min)	r_2 SD	r_{gen} (CTCs/hour)	r_{gen} Err	$t_{1/2}$ (sec)	$t_{1/2}$ Err
SCLC	Autochthonous	SR7401	1	0.06	1.57	5.90	0.82	0.49	0.03	4282.02	1232.03	97.28	15.85
		SR7464	2	0.06	1.51	17.10	0.61	1.22	0.10	14352.69	1599.76	80.08	7.92
		SR7402	3	0.06	1.59	11.85	0.51	0.48	0.09	17624.38	3531.58	46.10	8.88
		SR6458	4	0.06	1.52	17.67	0.20	0.97	0.13	19253.09	2638.61	61.24	8.73
		SR7391	5	0.06	1.46	25.20	0.40	1.39	0.09	27380.58	1950.07	59.07	4.10
PDAC	Autochthonous	PC1630	1	0.06	1.11	0.46	0.05	0.04	0.00	287.75	64.13	80.87	14.49
	Organoid Neo	WFP8367	1	0.06	1.68	0.11	0.01	0.01	0.00	58.15	18.75	141.81	45.53
		WFP8387	2	0.06	1.46	0.45	0.03	0.05	0.01	237.30	70.26	128.41	38.32
		WFP8296	3	0.06	1.62	0.81	0.04	0.15	0.01	247.32	30.34	261.05	24.99
		WFP8297	4	0.06	1.65	78.49	6.55	6.82	0.25	53790.33	9264.76	108.81	10.16
		WFP8296	5	0.06	1.58	0.87	0.04	0.16	0.02	272.55	51.42	249.45	47.65
	Organoid	KM0275	1	0.06	1.75	1.04	0.04	0.08	0.02	783.59	181.48	104.19	24.97
		KM0275	2	0.06	1.59	0.68	0.03	0.06	0.01	421.89	71.41	115.71	18.65
		KM0285	3	0.06	1.78	1.36	0.42	0.14	0.02	804.21	515.79	137.86	47.16
		KM0292	4	0.06	1.80	17.09	0.92	3.53	0.22	4754.00	623.25	325.01	31.34
		GC538	5	0.06	1.62	0.79	0.33	0.15	0.08	250.32	258.75	253.07	202.60
		KM966	6	0.06	1.27	1.50	0.24	0.24	0.06	547.95	229.66	168.03	62.38
		GC532	7	0.06	1.36	1.26	0.16	0.26	0.01	351.97	94.08	244.00	45.37
		GC534	8	0.06	1.60	87.78	16.27	4.36	0.29	105701.94	39901.63	58.11	11.49
NSCLC	Autochthonous	MB11085	1	0.06	1.80	0.28	0.02	0.03	0.01	174.23	63.18	130.31	48.79
		MB11126	2	0.06	1.20	0.93	0.07	0.07	0.02	755.61	267.80	66.37	23.46
		MB11124	3	0.06	1.29	0.30	0.09	0.05	0.01	95.40	66.12	198.12	94.52
		MB11085	4	0.06	1.68	0.73	0.04	0.08	0.01	405.96	62.28	139.58	17.26
		MB10985	5	0.06	1.42	0.85	0.35	0.17	0.00	247.96	211.31	241.73	123.40

Supplementary Table 3 | Circulatory kinetics of CTCs in mice. Summary of the circulatory kinetics and the normalized shedding rates (to the primary tumors of origin) of CTCs in mouse models of SCLC, PDAC, and NSCLC.

	Rgen Range (CTCs/HR)	Half-life Range (seconds)	CTCs shed/gram tumor/day	Percent of primary tumor cells shed/day (avg)
SCLC	4,000-27,000	50-100	700,000	0.07%
PDAC	65-6,000	55-260	40,000	0.004%
NSCLC	100-800	60-260	20,000	0.002%

Supplementary Table 4 | Primer sequences for RNA-Seq. Template switching oligo (TSO); Primers for whole genome amplification (ISPCR)

Primer	Sequence
OligodT (5' → 3')	/5Biosg/AAG CAG TGG TAT CAA CGC AGA GTA CTT TTT TTT TTT TTT TTT TTT TTT TTT TTT TVN
TSO (5' → 3')	AAG CAG TGG TAT CAA CGC AGA GTA CAT rGrGrG
ISPCR (5' → 3')	AAG CAG TGG TAT CAA CGC AGA GT
