# Supplementary material for: Measuring kinetics and metastatic propensity of CTCs by blood exchange between mice
Source: Nat Commun. 2021 Sep 28;12:5680. doi: 10.1038/s41467-021-25917-5 (PMC8479082; doi:10.1038/s41467-021-25917-5)
Supplement: Supplementary file 2 — Reporting Summary [file 41467_2021_25917_MOESM2_ESM.pdf]

nature portfolio

Corresponding author(s): Scott Manalis

Last updated by author(s): Jul 21, 2021

# Reporting Summary

Nature Portfolio wishes to improve the reproducibility of the work that we publish. This form provides structure for consistency and transparency in reporting. For further information on Nature Portfolio policies, see our Editorial Policies and the Editorial Policy Checklist.

## Statistics

For all statistical analyses, confirm that the following items are present in the figure legend, table legend, main text, or Methods section.

| n/a | Confirmed | |
|---|---|---|
| ☐ | ☒ | The exact sample size (*n*) for each experimental group/condition, given as a discrete number and unit of measurement |
| ☐ | ☒ | A statement on whether measurements were taken from distinct samples or whether the same sample was measured repeatedly |
| ☐ | ☒ | The statistical test(s) used AND whether they are one- or two-sided<br>*Only common tests should be described solely by name; describe more complex techniques in the Methods section.* |
| ☒ | ☐ | A description of all covariates tested |
| ☐ | ☒ | A description of any assumptions or corrections, such as tests of normality and adjustment for multiple comparisons |
| ☐ | ☒ | A full description of the statistical parameters including central tendency (e.g. means) or other basic estimates (e.g. regression coefficient) AND variation (e.g. standard deviation) or associated estimates of uncertainty (e.g. confidence intervals) |
| ☐ | ☒ | For null hypothesis testing, the test statistic (e.g. *F*, *t*, *r*) with confidence intervals, effect sizes, degrees of freedom and *P* value noted<br>*Give P values as exact values whenever suitable.* |
| ☒ | ☐ | For Bayesian analysis, information on the choice of priors and Markov chain Monte Carlo settings |
| ☐ | ☒ | For hierarchical and complex designs, identification of the appropriate level for tests and full reporting of outcomes |
| ☐ | ☒ | Estimates of effect sizes (e.g. Cohen's *d*, Pearson's *r*), indicating how they were calculated |

*Our web collection on statistics for biologists contains articles on many of the points above.*

## Software and code

Policy information about availability of computer code

| | |
|---|---|
| Data collection | LabVIEW 2017 Version 17.0, NIS-Elements Advanced Research version 4.20 |
| Data analysis | MATLAB R2017b Version 9.3.0.713579, R version 3.6.2, RStudio Version 1.2.1335, Microsoft Excel 2016, GraphPad Prism 9, Bowtie 2, Seurat 3.0, Vevo Lab 5.6.0 (FUJIFILM VisualSonics)<br><br>Code Availability Statement:<br>CTC processing code can be found at https://github.com/amiller92/CTCPeakAnalysis. Additional code will be available upon request. |

For manuscripts utilizing custom algorithms or software that are central to the research but not yet described in published literature, software must be made available to editors and reviewers. We strongly encourage code deposition in a community repository (e.g. GitHub). See the Nature Portfolio guidelines for submitting code & software for further information.

## Data

Policy information about availability of data

All manuscripts must include a data availability statement. This statement should provide the following information, where applicable:

- Accession codes, unique identifiers, or web links for publicly available datasets
- A description of any restrictions on data availability
- For clinical datasets or third party data, please ensure that the statement adheres to our policy

Data Availability Statement:
The single-cell RNA sequencing data reported in this paper is deposited in the NCBI Sequence Read Archive (Accession Number- PRJNA670615). The data that support the findings of this study are available from the corresponding authors upon reasonable request.

This BioProject was made public on July 16, 2021

# Field-specific reporting

Please select the one below that is the best fit for your research. If you are not sure, read the appropriate sections before making your selection.

☒ Life sciences ☐ Behavioural & social sciences ☐ Ecological, evolutionary & environmental sciences

For a reference copy of the document with all sections, see nature.com/documents/nr-reporting-summary-flat.pdf

# Life sciences study design

All studies must disclose on these points even when the disclosure is negative.

| | |
|---|---|
| Sample size | No sample size calculation was performed. For blood exchange experiments, our goal was to have at least triplicate for each cancer type in order to perform statistical analysis. Based on availability of tumor-bearing mice, we were able to increase our cohort size in each of the cancer types. Furthermore, this number of replicates (14 for PDAC and 5 for SCLC) allowed us to delineate between PDAC and SCLC half-life time with a p value below 0.05 (p=0.0136). For all remaining studies, at least tripicate experiments were performed to allow for statistical analysis. |
| Data exclusions | No data was excluded |
| Replication | All attempts at replication were successful.<br><br>CTC dynamics measurements resulted from SCLC blood-exchange experiments that were performed on five pairs of mice from three different cohorts. The PDAC blood-exchange experiments were performed on 14 pairs of mice with three different cohorts of tumor models. The 5 NSCLC blood exchanges were from the same cohort, but all blood exchanges for all tumor models were performed on independent days.<br><br>The outgrowth of SCLC CTCs into distant metastases in originally-healthy recipient mice was confirmed in four different mice within two to three months post the blood exchange experiment. All of the mice that developed tumors were confirmed to have CTCs present in the blood (either by microscopy or detection in the CTC sorter)<br><br>The cell-line intravenous injection experiments (bolus and slow) were performed in triplicates on independent days to confirm the findings.<br><br>The minimal loss study (Supp 2b) was only performed once- no attempt at replication was made<br>The SMR experiment (Supp 4a/b) was only performed once- no attempt at replication was made |
| Randomization | Our animal experiments focused on identifying mice with a specific tumor burden that was assessed by IVIS, microCT, or ultrasound for the blood exchange experiments. No drug administration or efficacy studies were performed and therefore randomization was not necessary |
| Blinding | Blinding was not relevant to this study. It was essential that 1 tumor bearing and 1 healthy mouse be used for each exchange, so that could not be blinded. Data analysis was performed autonomously with Matlab, with minimal user input |

# Reporting for specific materials, systems and methods

We require information from authors about some types of materials, experimental systems and methods used in many studies. Here, indicate whether each material, system or method listed is relevant to your study. If you are not sure if a list item applies to your research, read the appropriate section before selecting a response.

## Materials & experimental systems

| n/a | Involved in the study | |
|---|---|---|
| ☐ | ☒ | Antibodies |
| ☐ | ☒ | Eukaryotic cell lines |
| ☒ | ☐ | Palaeontology and archaeology |
| ☐ | ☒ | Animals and other organisms |
| ☒ | ☐ | Human research participants |
| ☒ | ☐ | Clinical data |
| ☒ | ☐ | Dual use research of concern |

## Methods

| n/a | Involved in the study | |
|---|---|---|
| ☒ | ☐ | ChIP-seq |
| ☒ | ☐ | Flow cytometry |
| ☒ | ☐ | MRI-based neuroimaging |

## Antibodies

| | |
|---|---|
| Antibodies used | anti-mouse CD45-FITC from Invitrogen (Cat # 11-0451-82) |
| Validation | Validated in multiple publications for immunofluorescence (IF) of immune cells (Larmour et al, PloS One 2018; Moroishi et al, Cell |

Validation | 2016; Messaoudi et al, Nat Comm, 2015)

# Eukaryotic cell lines

Policy information about cell lines

Cell line source(s): Cell line established in the lab of Tyler Jacks from murine lymph node metastasis of authocthonous SCLC tumor (p53-/-; pten-/-; Rb-/-; TdTomato+/+)

Authentication: DNA sequencing (Dooley et al. Genes Dev. 2011)

Mycoplasma contamination: All cell lines tested negative for mycoplasma contamination

Commonly misidentified lines (See ICLAC register): No commonly misidentified cell lines were used in this study

# Animals and other organisms

Policy information about studies involving animals; ARRIVE guidelines recommended for reporting animal research

Laboratory animals:

For the SCLC model, Ptenflox/flox; Trp53flox/flox; Rb1flox/flox; Rosa26LSL-Luciferase/LSL-Luciferase mice were maintained on mixed C57BL/ 6;129/Sv background mice obtained from Jackson Laboratories (Dooley et al., 2011 and DuPage et al., 2009). Mice were infected with a viral Cre at 12-15 weeks of age and then monitored for tumor burden using bioluminescence imaging until the age of 36-40 weeks (or 5-6 months post intra-tracheal infection).

For the NSCLC model, KrasLSL-G12D/+; p53fl/fl; Rosa26LSL-tdTomato/LSL-Cas9 mice (C57BL/6) were infected with adenovirus expressing Cre recombinase around 8-10 weeks of age and monitored for tumor burden using microCT until the age of 26-30 weeks (about 4-5 months post tumor initiation)

For Autochthonous PDAC experiments, KPT (Kras LSL-G12D/+; Trp53 fl/fl; R26 LSL-TdTomato/LSL-TdTomato) mice on a C57BL/6 background were infected with viral Cre retrograde into the pancreatic duct around 6 weeks of age and monitored for tumor burden using ultrasound until the age of 14-18 weeks (2-3 months post tumor initiation)

For orthotopic organoid PDAC experiments, PT mice on a C57BL/6 background had KPT or KPmScarlet+neoantigen organoids transplanted into the pancreas around 6 weeks of age and monitored using ultrasound until the age of 14-18 weeks (2-3 months post tumor transplantation)

Both male and female animals were used in approximately equal numbers

Wild animals: Study did not involve wild animals

Field-collected samples: Study did not involve field-collected samples

Ethics oversight: All animal-based procedures were approved by the Massachusetts Institute of Technology Committee on Animal Care (CAC), Division of Comparative Medicine (DCM).

Note that full information on the approval of the study protocol must also be provided in the manuscript.
